# Predecting power transformer health index and life expectation based on digital twins and multitask LSTM-GRU model

**DOI:** 10.1038/s41598-024-83220-x

**Published:** 2025-01-08

**Authors:** Nora El-Rashidy, Yara A. Sultan, Zainab H. Ali

**Affiliations:** 1 Department of Machine Learning and Information Retrieval, Faculty of Artificial Intelligence, Kafrelsheikh University, El-Geish St, Kafrelsheikh, 33516 Egypt; 2 Department of Mechatronics, Faculty of Engineering, Horus University-Egypt, New Damietta, Egypt; 3 Department of Embedded Network Systems and Technology, Faculty of Artificial Intelligence, Kafrelsheikh University, El-Geish St, Kafrelsheikh, 33516 Egypt; 4 Department of Electronics and Computer Engineering, School of Engineering and Applied Sciences, Nile University, Giza, Egypt

**Keywords:** Power transformer, Transformer health index, Machine learning, LSTM_GRU, Digital twins, Engineering, Mathematics and computing, Nanoscience and technology

## Abstract

Power transformers play a crucial role in enabling the integration of renewable energy sources and improving the overall efficiency and reliability of smart grid systems. They facilitate the conversion, transmission, and distribution of power from various sources and help to balance the load between different parts of the grid. The Transformer Health Index (THI) is one of the most important indicators of ensuring their reliability and preventing unplanned outages. To this end, this study introduces a proposed new architecture called a Smart Electricity Monitoring System based on Fog Computing and Digital Twins (SEMS-FDT) for monitoring the health performance of transformers by measuring the THI rate in real time. The SEMS-FDT is specifically designed to enable the observation of the transformer’s health and performance purposes in real time. The study investigates the role of machine learning (ML) models, including traditional and ensemble methods, in predicting THI and LI (Heat Load Index) by exploring the use of the entire set of features and optimized feature subsets for prediction. To improve the forecasting prediction process and achieve optimal performance a novel multitasks LSTM_GRU model is also proposed. The experimental results demonstrate that there is a promising performance of 2.543, 0.13646, 0.0284, and 0.985 for MSE, MAE, MedAE, and R2 scores respectively. Moreover, the framework is extended by incorporating model explanations, which include global explanations, which provide insights based on the entire dataset, and local explanations, which offer instance-specific explanations. The integration of the proposed model and explainability features provides engineers with comprehensive outcomes regarding the model’s result.

## Introduction

### Overview and motivation

Power transformers are proper electrical devices to facilitate electrical power transmission from one circuit to another while maintaining the same frequency. They are essential for maintaining a stable and reliable power supply to homes, businesses, and other critical infrastructure. By prioritizing transformers’ condition assessment, power system operators can ensure the efficient and reliable operation of power transformers, ultimately leading to a robust and resilient power system. Practically, the longevity of a transformer is heavily dependent on the condition of its insulation system. Thermal stress, moisture, electrical stress, and contaminants are the seed factors not only leading to the degradation of the insulation system but also reducing dielectric strength and increasing the overall risk of faults and failures. Regular monitoring and maintenance of the insulation system are crucial to ensure the longevity and reliable operation of power transformers. This includes routine testing of the insulating oil to assess its quality and dielectric strength, as well as periodic assessment of the impregnated paper for signs of degradation^1^.

Electrical and chemical test techniques are essential diagnostic tools for assessing the condition of the oil insulation in power transformers. These tests help detect potential faults and identify the type of insulation degradation, allowing for timely maintenance and repairs. Dissolved Gas Analysis (DGA) is one of the most commonly used chemical methods for evaluating the insulation condition in power transformers. This technique involves analyzing the gases dissolved in the insulating oil to identify and quantify the presence of specific gas types. The presence and concentration of certain gases can indicate different types of insulation faults or abnormalities occurring within the transformer. During regular operation of the transformer, the low levels of concentrations of gases include hydrogen (${H}_{2}$), methane (${CH}_{4}$), acetylene (${C}_{2}{H}_{2}$), ethylene (${{C}_{2}H}_{4}$), ethane (${{C}_{2}H}_{6}$), carbon monoxide ($CO$), and carbon dioxide (${CO}_{2}$) may be released. However, in cases of thermal and electrical strains, these gases can be created in large concentrations during fault conditions. Thus, the DGA approaches are significant in assessing transformer performance by detecting the released concentrations to identify and mitigate potential issues^2,3^.

To prove the effectiveness of a power transformer, the Transformer Health Index (THI) is used to assess the overall transformer’s performance under different conditions^4,5^. THI is a powerful tool that not only detects the current conditions of the transformer but also furnishes valuable technical data for operators of utility systems. The THI considers various factors such as the transformer’s expected lifespan, the environment in which it operates, and relevant tests. By comprehensively assessing these factors, THI provides operators and maintenance personnel with valuable information for determining the transformer’s condition and making informed decisions about its future use and maintenance. The operation inside THI is mainly based on several tests including DGA, chemical and physical analyses of oil, visual inspection results, furan concentration measurements, and electrical measurement parameters.

There is a relationship between the THI, the expected lifetime of the transformer, and the final decision about maintenance and replacement, aiding in ensuring that the power system operates reliably and efficiently while minimizing downtime and costs. As obvious, THI states for power transformers correspond to typical limits, decisions, and expected lifetimes. THI is a comprehensive measure of a transformer’s overall condition and reliability, based on several factors including temperature, oil quality, and electrical insulation properties. In detail, when the state of the transformer’s THI is “Very Good” (85% or higher), it is expected to have a lifetime of over 15 years, and thus the decision is no maintenance is required. Similarly, if the state of THI is good (between 70 and 85%), the transformer lifetime is expected to last more than 10 years, and the final decision is no maintenance is required. If the state of THI is between “Very Good” and “Good” the expected lifetime is over 15 and 10 years, and thus the final decision is no maintenance is required. If the state of THI is between “Fair” and “Poor”, the final decision is that diagnostic testing or replacement planning is required. Otherwise, if the value of THI falls within “Very Poor” (less than 30%), the transformer must be taken out of service and replaced immediately.

Leveraging the power of Machine Learning (ML) and Deep Learning (DL) offer flexible and comprehensive ways to capture complex relationships and detect significant insights between variables and targets. Regarding predicting THI and LI of the transformant^6,7^. For example, Artificial neural network (ANN) and fuzzy systems utilized to calculate TH^8^. The same in^9^, However, these methods have not been utilized for calculating more than one task concurrently. Multitask learning is a promising technique to optimize several tasks concurrently. It occurred by exchanging several features between tasks^10^. By utilizing MTL, the model could be more robust and generalizable as it could explode several layers among the shared tasks. MTL also improves the model stability as each task works the regularization layer for the other task, improving the developed model’s overall accuracy. In this study, we work on two regression tasks. MTL is used to predict the transformer’s health index and life expectation simultaneously, which is considered critical information for engineers.

### Novelty and contributions

To address THI and the expected lifetime of the power transformer, the robustness architecture that supports integration between the smart grid and digital twin technology is proposed. Recently, the interpretation of power transformer incipient faults has gained a lot of attention, especially in the methods that support DGA and THI such as in^11–13^, and^14^. However, low accuracy of the diagnostic analysis is the dominant with these methods. The reason, among others, is the lack of a real-time monitoring system can reduce a delay in communication and decision-making, whether it is in providing the necessary maintenance of the transformer or the speed of its replacement. This study introduces a Smart Electricity Monitoring System based on Fog Computing and Digital Twins (SEMS-FDT) for monitoring transformer health performance by measuring the rate of THI. The design of SEMS-FDT enables the observation of the transformer’s health and performance purposes in real-time. The proposed architecture is deployed by three layers that can support state-of-the-art technologies called the Internet of Things (IoT) and smart grid, digital twins, and fog computing. The novelty of the proposed architecture lies in its unique structure and design that ensures robust operation and reliable streaming transmission that reflects on the performance of the prediction system.

The work in this study is divided into two phases: (1) introducing a new design that supports the integration between smart grid and digital twins to improve the overall real-time monitoring performance and (2) demonstrating multitask learning technique based on Long Short-Term Memory- Gated Recurrent Unit (LSTM- GRU) to improve accuracy in the prediction process and boost model stability as each task works the regularizationation layer for the other task. Certainly, multitask learning is a technique that allows a model to learn multiple related tasks simultaneously, leveraging the shared information between them to improve overall performance. When combined with LSTM and GRU, it can enhance the capabilities of the model to handle sequential data and perform multitask learning efficiently^15^.

LSTM and GRU are both types of recurrent neural networks (RNNs) that excel at processing and modeling sequential data. They are particularly effective in capturing long-term dependencies and handling variable-length input sequences. LSTM is known for its ability to mitigate the vanishing gradient problem and remember information for long periods, while GRU provides a simpler architecture with fewer parameters but comparable performance. By combining LSTM and GRU in a multitask learning framework, we can take advantage of their strengths and address different tasks simultaneously. The shared layers of the model, such as the LSTM and GRU layers, capture the common patterns and dependencies across the tasks, providing a form of regularization and enabling the model to generalize better^16,17^. The contributions of this study are summarized as follows:

i. Introducing real-time observation and control system with a unique design called SEMS-FDT for monitoring transformer health condition and measuring the value of THI.
ii. Collecting real-time streaming about energy consumption, power quality, health condition, and equipment performance using IoT-devices and digital twins. This data helps to speed up identifying inefficiencies, detecting faults or outages, and optimizing energy usage.
iii. Enhancing resiliency and fault detection using the integration of smart grid and digital twin technology. The digital twins install as software on transformers to detect anomalies, abnormal behavior, or potential failures that enable predictive maintenance and the timely replacement of faulty transformers. This enhances grid resiliency and reduces the risk of disruptions.
iv. Establishing a mathematical model allows system to evaluate the theoretical value of THI, dissolved gas index, oil-quality, and routine test.
v. Employing Recursive Feature Elimination (REF) as a technique to select the most significant features for our model. This step aimed to enhance the efficiency and effectiveness of the feature set used in t proposed model.
vi. Exploring the role of ML (traditional and ensemble) models in predicting THI and LI, with the whole features and optimized features.
vii. Proposing a novel multitask LSTM_GRU model that could predict THI, and LI concurrently in order to improve forecasting prediction and achieve optimal real time performance in the transformers. As well as, appling grid search, a systematic method, to explore various combinations of hyperparameters. This process allowed us to identify the optimal hyperparameter values that yielded the best results.
viii. Extending the framework by adding model explanations in terms of global explanation that provide explanation according to the whole dataset and local explanation that provide the explanation according to the instance. The proposed model and the explainability features utilized to provide the engineers with complete outcomes about the model results.

### Paper organization

The rest of this study is organized as follows: Section "Related work" reviews the related works and recent technology to evaluate the transformer health condition and expected lifetime. Section "Proposed smart electricity monitoring system based on fog computing and digital twins" introduces the real-time monitoring system called SEMS-FDT that works based on state-of the-art techniques. Section "Material and methods" demonstrates material and methods. Section "Proposed architecture (LSTM_GRU) model" provides details about applying proposed architecture LSTM_GRU Model. Section "Results and discussion" discusses experimental setting. Section "Explanation of proposed model" provides the results for the multitask learning technique based on LSTM-GRU. Section "Conclusion" wraps up the study with a brief overview of all previous sections.

## Related work

Traditional methods for calculating THI of power transformers involve assessing various parameters, including^18^:

i. Insulation resistance: to evaluate the strength of the transformer’s insulation, this method entails measuring the insulation resistance between the winding and the ground.
ii. Power factor: this approach involves monitoring the phase difference between voltage and current to determine the efficiency of the transformer.
iii. DGA: by analyzing the types and quantities of gases dissolved in the transformer oil, it can detect the presence of electrical or thermal faults.
iv. Furan analysis: this method involves measuring the concentration of furanic compounds in the transformer oil, which can indicate the degree of degradation of the insulation material.
v. Oil quality: to assess the quality of the transformer oil and identify any potential issues, this method involves analyzing its physical and chemical properties, including acidity, moisture content, and flash point.
vi. Visual inspection: it is conducted to detect any signs of damage or wear, such as cracks, corrosion, or oil leaks.

While these methods have been used for many years and can provide valuable information about the transformer’s health, they require manual data collection and analysis, which can be time-consuming and prone to errors. Additionally, they may not be able to detect subtle changes in the transformer’s condition that could indicate potential issues. The scoring-weighting method, also known as the weighted sum, is a frequently used approach for computing THI of power transformers. This procedure entails the comparison of each parameter with a standardized scoring table, assigning weights based on their importance, and combining the scores into a single index. However, THI’s reliance on weighted measurement data and scores from various parameters can pose a challenge in dealing with associated uncertainties^19^.

Recently, the field of THI has witnessed the emergence of innovative approaches that rely on ML algorithms to analyze big data. THI approach based on a neuro-fuzzy model is proposed in^20^, which utilized DGA, Data obtained from furan and oil tests to evaluate the health status of several transformers in operation. It combines the learning and adaptive aspects of neural networks with the reasoning and decision-making abilities of fuzzy logic; however, the developed neuro-fuzzy model exhibited an accuracy of only 56.3% due to the scarcity of available data. Markov Model is presented in^21^. It is a predictive approach that leverages THI to estimate the future states of transformers. It relies on a probability-based decision process, wherein maintenance decisions depend on the current performance of assets. In this approach the stochastic model can quantify the uncertainty associated with the deterioration process of equipment, making it more flexible.

In^22^, The evaluation of transformer condition based on THI was carried out utilizing an Artificial Intelligence (AI) system that integrated fuzzy-based support vector machines (SVM), which considered a range of factors, including industry standards and expert opinions from utilities. The underlying principle of this approach is that the measurement data used for calculating the THI is frequently incomplete or uncertain. To address this uncertainty, fuzzy logic is employed to map input variables to a linguistic variable that represents the degree of membership in a specific class or category. However, SVM can classify samples into certain categories, it is unable to diagnose faults associated with specific information. Moreover, In the case of multiple classification problems, the model may result in overlapping or inseparable classifications.

The calculation of THI for transformers was carried out by employing an artificial intelligence-based general regression neural network (GRNN), relying on a four-class transformer condition classification system (very poor, poor, fair, and good) presented in^23^. The GRNN algorithm can handle non-linear input–output relationships, making it well-suited for complex transformer health assessment tasks. However, its accuracy cannot be considered reliable. In^24^, A customized approach for calculating THI of transformers was proposed, utilizing Bayesian networks and artificial intelligence techniques. This approach enabled the quantification of the contribution of different parameters to the HI, using score probability and population failure statistics. It is trained using a dataset of historical transformer data, and the input variables are mapped to a high-dimensional feature space that is used to calculate THI. The Bayesian network algorithm can handle uncertainty and incomplete data by considering prior knowledge and incorporating new data as it becomes available, but this model is inadequate for handling power transformers with varying voltages due to its incomplete consideration of state parameters.

A novel method for assessing the health condition of transformers is introduced in^25^, which combines a principal component analysis (PCA) and an analytical hierarchy process (AHP) with an expert empirical formula. PCA is a method that can reduce the dimensionality of a dataset that has a high number of correlated variables. Through the process of transforming the original variables that are correlated with each other into a new set of uncorrelated variables, referred to as principal components (PCs), PCA maintains the original data variation. The drawback of AHP is that the construction of the judgment matrix relies heavily on empirical judgment, making the importance between parameters less objective. This renders AHP unsuitable for directly calculating the evaluation value of the original data.

An artificial neural network technique is provided in^26^. The distribution of knowledge in an Artificial Neural Network (ANN) is discrete and follows sample learning. Despite being a powerful knowledge acquisition tool, ANNs also have several limitations. For instance, in cases where the difference between training samples and fault samples is significant, the reasoning of the ANN in concluding may become questionable. Moreover, to guarantee reliability, it is crucial to train and test the networks on a sufficiently large dataset.

## Proposed smart electricity monitoring system based on fog computing and digital twins

THI is a metric used to assess the overall health and condition of the transformer’s performance. It provides valuable insights into the transformer’s performance, reliability, and potential risks. THI considers various parameters and factors that can influence the transformer’s operational efficiency and longevity. THI can be further enhanced and leveraged by consolidating digital twins with IoT technology. Digital twins refer to virtual replicas of physical assets or systems that enable real-time monitoring, analysis, and simulation of their behavior and performance. When combined with IoT, which involves connecting physical devices and sensors to the internet, THI can benefit from increased data availability and advanced analytics capabilities. This study introduces the proposed architecture called a Smart Electricity Monitoring System based on Fog Computing and Digital Twins (SEMS-FDT) for monitoring transformer health performance by measuring the rate of THI. The SEMS-FDT is specifically designed for enabling the observation of the transformer’s health and performance purposes in real-time. The proposed SEMS-FDT is deployed by three-layer are IoT and smart grid, digital twins, and fog computing.

**Fig. 1:**
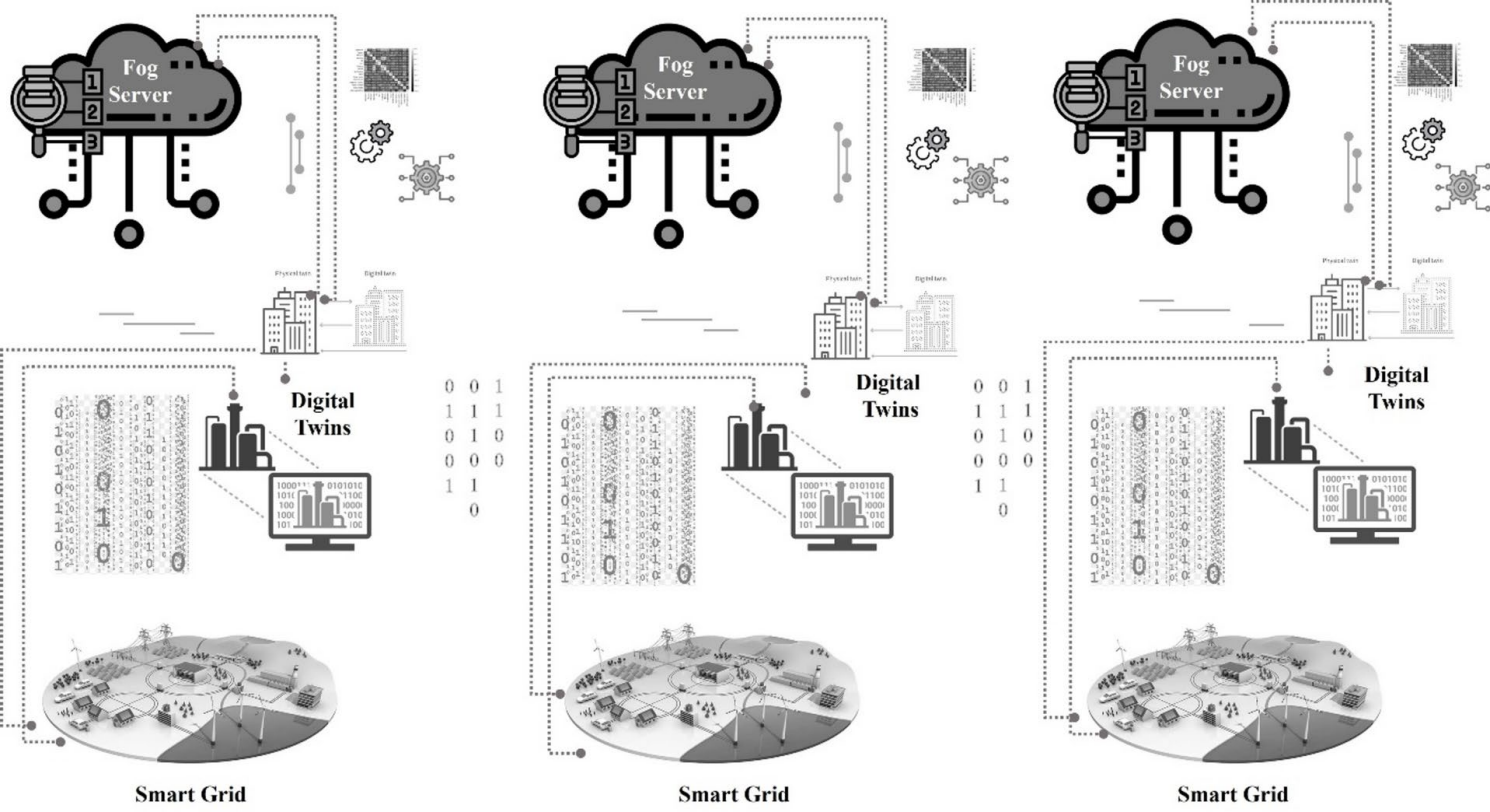
The design of SEMS-FDT.

The amalgamation of IoT with the smart grid has revolutionized the way electricity is generated, distributed, and consumed. As shown in Fig. 1, the first layer includes a smart grid and IoT devices. The smart grid refers to an intelligent electrical grid that incorporates digital communication and automation technologies to enhance efficiency, reliability, and sustainability. Transformers are essential components of the electrical grid that step-up or step-down voltage levels to facilitate the transmission and distribution of electricity. They ensure that electricity is delivered at the appropriate voltage levels for various applications. However, traditional transformers often lack advanced monitoring and control capabilities. For that, IoT technology with sensors and intelligent devices is integrated with the smart grid. IoT in the smart grid involves connecting various transformers, electrical sources, and equipment throughout the electrical grid to gather real-time data and enable remote monitoring and control. These IoT-enabled devices can include smart meters, sensors on power lines, distribution transformers, and even consumer appliances. This interconnected network of devices forms the foundation of a smart grid ecosystem. IoT-enabled devices also enable load management, where appliances or equipment can be controlled remotely to optimize energy consumption. Maximizing resource utilization is the benefit of IoT technology, which can remotely adjust transformer settings, optimize loading, and balance power flow based on real-time data. This leads to improved grid stability, reduced losses, and increased energy efficiency.

### Network model

Grid network is represented using an undirected graph ${\varvec{G}}=({\varvec{V}},{\varvec{E}},{\varvec{T}}{\varvec{H}}{\varvec{I}})$. Here, the $({\varvec{V}}$) is the participated items in the grid network including transformers, so, ${\varvec{V}}=\{{{\varvec{s}}}_{1},{{\varvec{s}}}_{2},{{\varvec{s}}}_{3},\dots {{\varvec{s}}}_{{\varvec{n}}}\}$. An (${\varvec{E}})$ is the quality of link that established between sender $\left({\varvec{s}}\right)$ transformer and receiver $\left({\varvec{D}}{\varvec{T}}\right)$ digital twin device. The $({\varvec{T}}{\varvec{H}}{\varvec{I}})$ is the required value that needs to calculate theoretical health index of transformer.

For enhancing grid resilience and elevating system performance in detecting transformer anomalies, faults, or potential irreparable failures in real-time, the concept of digital twins has emerged as the second layer in the proposed SEMS-FDT. In the context of the smart grid, transformers play a crucial role, they are not just passive components but become intelligent assets that contribute to grid optimization and efficiency. In the proposed SEMS-FDT, transformers are equipped with IoT-controller and communication devices that enable real-time monitoring of their operational parameters via WiFi module ESP8266 Arduino ensuring effective internet connectivity range in the communication operation. These sensors can measure variables such as temperature, oil quality, load conditions, and DGA levels. The collected data is transmitted to a central monitoring system, allowing utilities to have better visibility and control over transformer performance.

The layer of the digital twin acquires the data about transformers from the IoT-controller in this case, the collected data is about DGA, which contains the information of gases, oil-paper isolator, and oil quality allows the digital twin layer to make informed decisions about maintenance, asset replacement, and overall grid reliability. Table 1 shows the types of data collected from transformers.

**Table 1:** Types of data collected from transformers.

Data type	Contain
DGA	${H}_{2}$
	$${O}_{2}$$
	$${N}_{2}$$
	$${CH}_{4}$$
	$${C}_{2}{H}_{2}$$
	$${C}_{2}{H}_{4}$$
	$${C}_{2}{H}_{6}$$
	$${CO}_{2}$$
	$$CO$$
Oil-quality	Dielectric strength
	Contaminant levels
	DGA
	Moisture content
	Oxidation and aging

Digital twins can continuously monitor the levels of dissolved gases in a system. This data is then compared against predefined thresholds to determine if any adjustments are necessary. Once a digital twin identifies that the threshold of dissolved gases has been exceeded or is approaching critical levels, it can trigger automatic adjustments to mitigate the issue. Digital twins are supported with feedback control systems that automatically adjust operational parameters to maintain dissolved gas levels within the desired range. According to the recommended gas values in Table 2, the layer of digital twins’ help optimizes system performance by adjusting thresholds to maintain the ideal balance of dissolved gases, ensuring efficient operation and longevity.

**Table 2:** Thresholds and weight of dissolved gases index^18^.

Gas	Thresholds ($\mu L/L$)		Weight (${W}_{i}$)
	High	Low	
${H}_{2}$	50	150	2
${CH}_{4}$	30	130	3
${C}_{2}{H}_{6}$	20	90	3
${C}_{2}{H}_{4}$	60	280	3
${C}_{2}{H}_{2}$	0	5	5
$CO$	400	600	1
${CO}_{2}$	3800	14,000	1

### Initial THI calculation

Power transformers usually adopt oil quality measurements. Oil-paper insulation is a common type of insulation used in power transformers. It consists of a combination of mineral oil and cellulose paper, providing electrical insulation and thermal stability. Monitoring the health of this insulation is crucial for ensuring the reliable operation of transformers. The insulating paper serves the secondary purpose of mechanical support in addition to its primary role as transformer insulation. Thermal stress causes the insulating paper to deteriorate over time. Insulating paper is made up primarily of cellulose, which ages by a process known as pyrolysis, which is a heat-chemical fracture reaction. The degree of polymerization (DP) value of the insulating paper will then drop because of pyrolysis. This DP value represents the mechanical strength of the transformer and is frequently used to describe the life of the transformer. A transformer’s initial DP value exceeds 1200 when it is first put into service; this value drops to 250 when the transformer approaches the end of its useful life. Even though the insulation performance of the insulating paper appears to be good when the DP value is less than 250, its mechanical strength has been severely diminished, leading to insufficient antishort circuit ability of the transformer, indicating that the transformer’s life has come to an end. According to [Z2], the value of DP can be calculated as follows:

1 $$\frac{1}{{DP}_{t}}-\frac{1}{{DP}_{o}}=\frac{{\varepsilon }_{10}}{{\varepsilon }_{2}}\left(1-{e}^{-{\varepsilon }_{2}t}\right)$$

where the whole DP value is equal the difference between the ${{\varvec{D}}{\varvec{P}}}_{{\varvec{t}}}$ denotes the degree of polymerization in a certain time ${\varvec{t}}$ and the initial value of DP called ${{\varvec{D}}{\varvec{P}}}_{{\varvec{o}}}$. The ${{\varvec{\varepsilon}}}_{10}$ and ${{\varvec{\varepsilon}}}_{2}$ are the constants values. The term ${{\varvec{e}}}^{-{{\varvec{\varepsilon}}}_{2}{\varvec{t}}}$ represents the thermal aging of the oil-paper isolation. According to Eq. (1), the theoretical value of transformer health index ${{\varvec{T}}{\varvec{H}}{\varvec{I}}}_{{\varvec{i}}}$ can be calculated as follows:

2 $${THI}_{i}={THI}_{o}-{e}^{\beta \times {T}^{o}}$$

where ${{\varvec{T}}{\varvec{H}}{\varvec{I}}}_{{\varvec{i}}}$ denotes the theoretical value of transformer health index. ${{\varvec{T}}{\varvec{H}}{\varvec{I}}}_{{\varvec{o}}}$ is the initial value of the theoretical value of transformer health index. The term ${{\varvec{e}}}^{{\varvec{\beta}}\times {\varvec{T}}}$ includes ${\varvec{e}}$ denotes the thermal aging of the oil-paper isolation, ${{\varvec{T}}}^{{\varvec{o}}}$ denotes the transformer operating time, and ${\varvec{\beta}}$ denotes the thermal aging coefficient adjusted with 0.5 and it can be determined as follows:

3 $$\beta = \frac{ln\left(\frac{{THI}_{i}}{{THI}_{0}}\right)}{lifetime}$$

where ${\varvec{l}}{\varvec{i}}{\varvec{f}}{\varvec{e}}{\varvec{t}}{\varvec{i}}{\varvec{m}}{\varvec{e}}$ denotes the expected lifetime of the transformer to work in good health condition without deterioration, it is related to the values of ${{\varvec{T}}{\varvec{H}}{\varvec{I}}}_{{\varvec{o}}}$ and ${{\varvec{T}}{\varvec{H}}{\varvec{I}}}_{{\varvec{i}}}$.

**Fig. 2:**
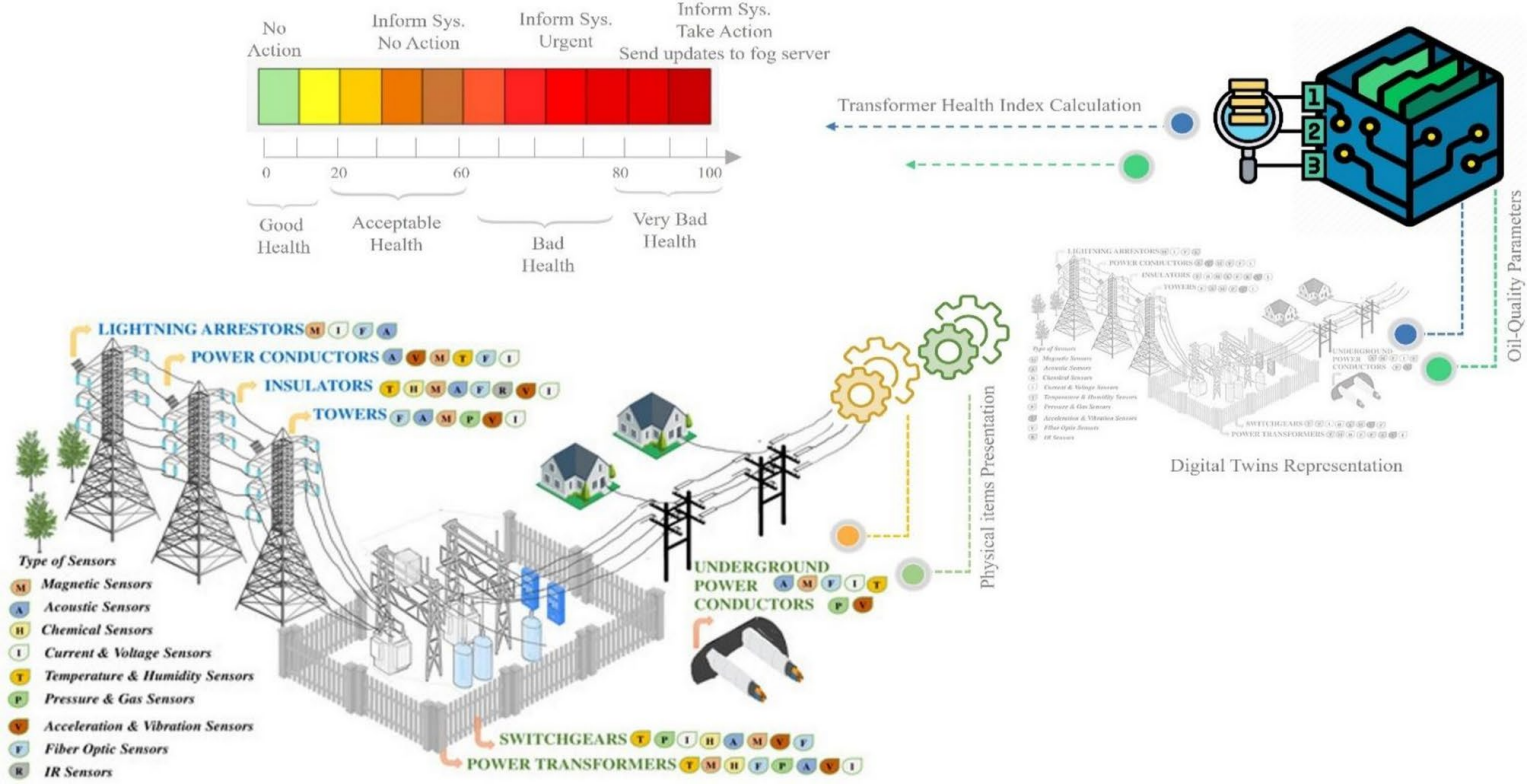
The operation of THI calculation using digital twins.

In the proposed SEMS-FDT, the theoretical transformer index assessment is adjusted between 0 to 100 instead of the traditional adjustment from 0 to 10 to add more flexibility in assigning weights to different diagnostic parameters and evaluating the overall health of the oil-paper insulation. This modification allows the digital twin to provide a more detailed and nuanced representation of the insulation’s condition, enabling maintenance teams to make informed decisions based on the severity of the health index value. As shown in Fig. 2, the range from 0 to 20 indicates that the transformer is very healthy, the range from 21 to 60 indicates that the transformer performance is not very good but still at the acceptable level. The range from 61 to 80 indicates that the transformer performance is bad health, and the electrical system needs intervention to restore the transformer to a stable state, more than 80 indicates that the transformer is already in the very bad condition thus the probability of f failure is very high. The learning technique in the digital twin layer prevents entry to the failure stage by sending an early warning message to all controllers stating the need to intervene to replace or even maintain the transformer. All updates about this network will be sent to the fog server for local storage. The benefits of adding fog technique in this proposed scenario can be confined to two directions: (i) reducing network bandwidth usage, thus increasing the rate of a successful data packet over the internet, and (ii) speeding up the decision-making because of the final decision will be made locally without the need to travel to the cloud server.

### Dissolved gas index (DGI)

Dissolved Gas Index (DGI) is a numerical value that represents the severity of the gas levels detected in the insulating oil of a transformer. As shown in Table 2, DGI is calculated based on the concentration of different gases dissolved in the transformer oil. These gases are produced because of various fault conditions within the transformer, such as overheating, insulation degradation, or electrical arcing. Each gas has a specific relationship with different types of faults or abnormalities occurring in the transformer. By monitoring the levels of these gases, experts can identify potential issues and take appropriate preventive or corrective measures to maintain the transformer’s health. The DGI can be formulated as follows:

4 $$DGI= \frac{{\sum }_{i=0}^{n}{G}_{|value}\times {W}_{i}}{{\sum }_{i=0}^{n}{ W}_{i}}$$

where term ${\sum }_{{\varvec{i}}=0}^{{\varvec{n}}}{{\varvec{G}}}_{|{\varvec{v}}{\varvec{a}}{\varvec{l}}{\varvec{u}}{\varvec{e}}}$ presents the scale of gases in DGI in our proposed architecture the measurements between 0 to 8 because of the number of gases is nine. ${{\varvec{G}}}_{|{\varvec{v}}{\varvec{a}}{\varvec{l}}{\varvec{u}}{\varvec{e}}}$ denotes the grades value of each gas that can be calculated based on the value in Table 2. The formula of ${{\varvec{G}}}_{|{\varvec{v}}{\varvec{a}}{\varvec{l}}{\varvec{u}}{\varvec{e}}}$ can be expressed mathematically by:

5 $${G}_{|value}= \left\{\begin{array}{cc}0 & 0\preccurlyeq \omega \prec a\\ 50+\text{sin}(\frac{\pi }{b-a}(\omega -\frac{b+a}{2}))& a\preccurlyeq \omega \preccurlyeq b\\ 100 & b\prec \omega \end{array}\right.$$

where ${\varvec{a}}$ and ${\varvec{b}}$ are the thresholds lower band and upper band for each gas.

### Oil-quality index

The Oil-Quality Index (OQI) is also another significant indicator to assess the THI. The factor of OQI plays a crucial role in THI calculation. Regular monitoring of the oil quality is essential for preventive maintenance and to detect potential problems before they escalate. By assessing the oil quality and monitoring the HI, maintenance teams can make informed decisions regarding oil filtration, replacement, or other necessary actions to ensure the transformer’s optimal performance and longevity. According to Table 3, the thresholds of oil-quality is defined.

**Table 3:** Thresholds of each affected factor in OQI ^20^.

Factor	Thresholds ($\mu L/L$)		Weight (${W}_{i}$)
	High (b)	Low (a)	
Breakdown voltage	40	60	3
Acid value	0.01	0.5	1
Water content	10	30	3
Dielectric loss ratio	0.2	4	4

According to Eq. 5, the formula of OQI can be expressed as follows:

6 $$OQI= \frac{{\sum }_{i=0}^{n}{G}_{|value }\times {W}_{i} }{{\sum }_{i=0}^{n}{W}_{i}}$$

where ${\sum }_{{\varvec{i}}=0}^{{\varvec{n}}}{{\varvec{G}}}_{|{\varvec{v}}{\varvec{a}}{\varvec{l}}{\varvec{u}}{\varvec{e}}\boldsymbol{ }}$ is the scale of factor ${\varvec{i}}$ for the OQI from 0 to ${\varvec{n}}=4$, the threshold of the ${{\varvec{G}}}_{|{\varvec{v}}{\varvec{a}}{\varvec{l}}{\varvec{u}}{\varvec{e}}}$ can be determined based on the upper limit (b) and lower limit (b) as mentioned in Table 3.

### Routine test calculation

Routine testing and the health index serve different purposes in transformer condition assessment. Routine testing focuses on defect detection, while the health index provides a comprehensive description of the transformer’s condition. By leveraging both approaches, maintenance programs in the layer of digital twins can effectively manage and optimize their transformer assets, ensuring reliable and efficient operation in the long run.

Routine testing is performed to identify any defects or abnormalities in a transformer. The primary objective is to detect potential issues that may impact the transformer’s performance or lead to failures. By conducting routine tests, technicians and engineers can pinpoint problems early on and take appropriate measures to prevent further damage or downtime. The specific test items carried out during routine testing may vary based on the site’s conditions and the availability of testing resources. In this study, the routine test calculations are dependent on nine-factor that listed in Table 4.

**Table 4:** List of routine tests.

#	Routine test
1	Short circuit test
2	Open circuit test
3	Winding resistance test
4	Transformer vector group test
5	Measurement of insulation resistance
6	Tests on on-load tap-changer
7	Dielectric tests
8	Transformer ratio test
9	Oil pressure test

The formula of routine test can be expressed mathematically as follows:

7 $${R}_{|test}=\frac{{\sum }_{i=1}^{n}{k}_{i}DGI}{{\sum }_{i=1}^{n}{k}_{i}}$$

where ${{\varvec{R}}}_{|{\varvec{t}}{\varvec{e}}{\varvec{s}}{\varvec{t}}}$ is the routine test of the transformer. The scale of tested in this case from 1 to 9, so ${\varvec{i}}$ is started with 1 to ${\varvec{n}}=9$ as shown in Table 4. ${{\varvec{k}}}_{{\varvec{i}}}$ is a constant that used to calculate weight coefficient for each test in the case of ${\varvec{D}}{\varvec{G}}{\varvec{I}}$, the ${{\varvec{k}}}_{{\varvec{i}}}$ equal 10^20^. ${\varvec{D}}{\varvec{G}}{\varvec{I}}$ is the dissolved gas index of the transformer.

### Final THI calculation

The final THI is designed to provide a holistic description of the transformer’s condition. Rather than focusing solely on defect detection, THI considers various factors and parameters to assess the overall health and performance of the transformer. The final THI incorporates data from routine tests, as well as additional information such as operational history, maintenance records, and environmental factors. By analyzing these inputs, the health index assigns a numerical value or rating that indicates the transformer’s condition. This allows digital twins layer to prioritize their efforts and allocate resources effectively. The final value of THI can be determined as follows:

8 $$THI=\left(A \times {THI}_{i}\right)+\left(B\times {THI}_{1}\right)$$

where ${\varvec{A}}$ and ${\varvec{B}}$ are constant value can be assigned as 0.4 and 0.5 respectively. ${{\varvec{T}}{\varvec{H}}{\varvec{I}}}_{{\varvec{i}}}$ is the theoretical value of transformer health index and can be calculated based on Eq. 2. The ${{\varvec{T}}{\varvec{H}}{\varvec{I}}}_{1}$ is the index value can be calculated based on the specific tests of the transformer, in this case, the specific tests are ${\varvec{D}}{\varvec{G}}{\varvec{I}}$ in Eq. 4 and ${\varvec{O}}{\varvec{Q}}{\varvec{I}}$ in Eq. 6.

## Material and methods

### Dataset

The calculation of THI depends on different calculations are: (1) DGA which relies on nine gases such as ${H}_{2}$, ${O}_{2}$, ${N}_{2}$, ${CH}_{4}$, ${C}_{2}{H}_{2}$, ${C}_{2}{H}_{4}$, ${C}_{2}{H}_{6}$, ${CO}_{2}$, and $CO$. (2) the quality of transformer oil is assessed based on dielectric rigidity, water content, and interfacial tension. Dielectric rigidity, gauged by breakdown voltage (BDV), reveals moisture or contaminants that can impede insulation and jeopardize transformer health. Moisture in oil degrades dielectric properties over time, necessitating regular BDV monitoring for transformer reliability. Water in oil affects core insulation, as paper absorbs water, diminishing its insulating capacity and lifespan. Oil temperature influences water solubility, crucial when collecting samples for testing. Oxidation generates acids in oil, heightening water solubility, accelerating degradation through acid–water interactions.

The interfacial tension, which is quantified in units of Dyne/cm or milli-Newton/meter, provides a measurement of the molecular forces of attraction between water and oil at their interface. This property is a valuable indicator of the presence of polar contaminants and decay products in transformer oil, as they can significantly affect the interfacial tension of the oil. In general, high interfacial tension is a characteristic of good-quality, new oil, whereas a decrease in interfacial tension over time may indicate the presence of contaminants or degradation products. This parameter is particularly useful in identifying the presence of polar contaminants, which are known to significantly impact the performance and longevity of transformer oil.

The transformer power factor is taken into consideration. It indicates the dielectric loss of oil, and it is commonly used for insulating oil approval and scheduled maintenance. Its power factor equals the cosine of the phase angle between the supplied AC voltage and the produced current. Despite extensive investigation, the source of corrosive sulfur in oil is yet to be definitively established, although it is widely believed to be a byproduct of the refining process. When corrosive sulfur, primarily dibenzyl disulfide (DBDS), in the insulation oil, interacts chemically with copper conductors, it leads to the formation of copper sulfide. This chemical reaction significantly modifies the dielectric characteristics of paper, and the accumulation of copper sulfide on the paper surface has a detrimental impact on the electric field. This can reduce insulating resistance, accelerate the aging of insulating materials, and even cause direct breakdown.

To implement the proposed technique, a dataset consisting of 940 transformer records was collected, which included information on transformers with varying ages, operating conditions, and health statuses. To ensure the reliability and generalizability of the model, the dataset was partitioned into two subsets: 80% of the data was reserved for training the model, while the remaining 20% was used for testing and validating the model’s performance. This approach ensured that the model was trained on a diverse range of transformer data, enabling it to capture the complex relationships between various parameters and their impact on transformer health. The summary statistics of a dataset include the count, mean, standard deviation (std), minimum, maximum, and percentiles (25%, 50%, and 75%), which provide information on the central tendency, variability, range, and distribution of the data is shown in Table 5.

**Table 5:** Summary description of the dataset’s key features.

	Count	Mean	Std	Min.	25%	50%	75%	Max.
Hydrogen	940	404.3	2001.1	− 0.3	3.9	9	9	23,349.1
Oxigen	940	8357.4	14,156.7	57	493	3810	3810	249,900
Nitrogen	940	47,759.6	13,753.1	3600	41,700	49,100	49,100	85,300.1
Methane	940	79.7	489.1	− 0.2	2	3.1	3.1	7406
CO	940	244	237.1	10	66	150.5	150.5	1730.1
CO_2_	940	1816.4	2255.6	48	641	1125	1125	24,900
Ethylene	940	162.9	1323.1	− 0.3	0	3	3	16,684.1
Ethane	940	81.9	342.4	− 0.3	0	4	4	5467
Acethylene	940	91.5	644	− 0.3	0	0	0	9740
DBDS	940	17	46.7	− 0.3	0	0	0	227
Power factor	940	1.9	6.1	− 0.1	0.6	1	1	73.2
Interfacial V	940	38.4	6.2	21	32.2	39.1	39.1	57
Dielectric rigidity	940	53.5	6.5	27	50.9	54	54	75.3
Water content	940	16.3	17.1	− 0.1	5	11.9	11.9	183
Health index	940	27.5	17.7	13.1	13.4	13.5	13.5	95.3
Life expectation	940	33	17.8	5.8	18.9	32	32	51.2

### Statistical significance test

In this study, we depend on both classifier evaluation metrics and statistical tests to ensure the model performance and check if is a significant difference between classifiers’ performance. Firstly, we used the Wilcoxon signed rank test developed by Demsar^21^. to compare the performance of all algorithms. This nonparametric test counts the Number of ties and wins obtaiithm. An algorithm is considered statistically better if it has more wins than ties. Next, the Friedman test was utilized to compare all algorithms^22^. Friedman is a nonparametric test that measures ANOVA, it could determine whether there was a significant difference between all utilized algorithms, but it did not identify the best algorithms. Therefore, to rank all utilized algorithms and choose the best one, we employed the Nemenyi test^23^ and calculated the average rank for each classifier. When comparing several models against each other, the results can be plotted using a critical distance diagram. This diagram shows the critical distance of all classifiers according to the average rank of the Nemenyi test and sepcify the best one.

### Multitask learning (MTL)

Multitask Learning (MTL) is an up-and-coming area of ML and DL that aims to leverage the valuable data from multiple learning challenges to develop a more suitable learner for each task^24^. When multiple tasks are learned together, they tend to outperform the performance achieved by learning them separately, both in terms of empirical and theoretical metrics, provided that the tasks are connected, or at least a subset of them are connected^25,26^. MTL can be considered a means for robots to mimic human learning behavior, as humans transfer information from one activity to another when these tasks are related. The main advantage of MTL is to undertake multiple learning tasks simultaneously, as the knowledge gained from one task can be applied to other related tasks^27^. Suppose we have n supervised learning task ti for I from 1 to n, each task has its dataset D (x,y) , where each x lies in diemnsional and y is the associated label for x. Therefore, for each task, there exist x n instance and the associated labels. MTL aims to make m tasks for m training data, such that F approximates y. After that, the developed function is utilized to predict the labels of the unseen data.

#### Long short-term memory (LSTM)

**Fig. 3:**
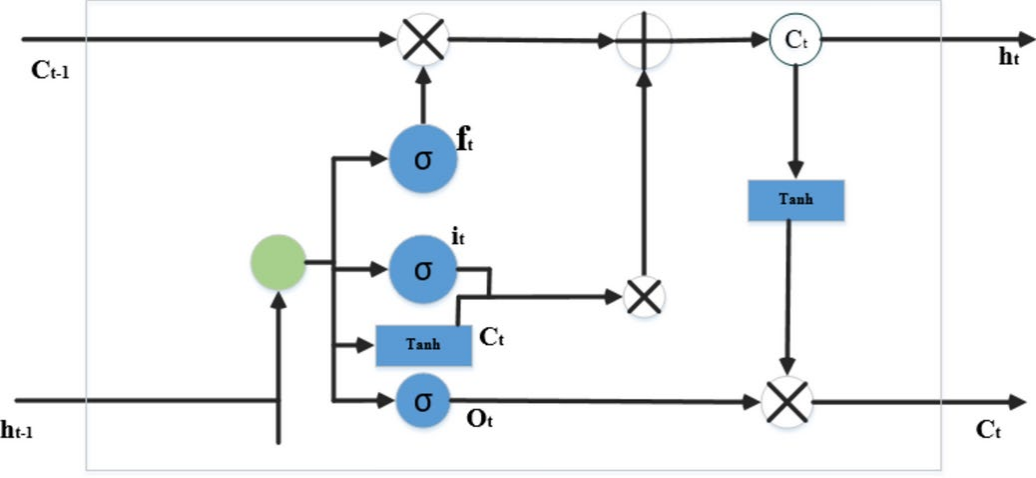
LSTM Cell structure.

LSTM is an enhanced version of a Recurrent neural network (RNN) mainly concerned with time series data. The general framework of LSTM includes three main gates (input gate, output gate, and forget gate). In addition to cell state, which is used to memorize the flow of information between output and forget gate) First, the input vector $${x}_{t}$$ combined with the previous hidden ($${h}_{t-1})$$ and bais $${b}_{f}$$. This is used to generate the forget gate activation function (see Eq. 9) equation of the LSTM model listed in the following equations. The activation vector of input as well as the update gate calculated by considering the input and the $${h}_{t-1}$$. Equation 10 shows the relationship of inputs with weights. Equations 11 and 12 show the activation vector of the output gate and the cell. Figure 3 details the LSTM cell structure. The calculation of hidden state is calculated in Eq. 13. Combine the last cell with the activation of the output gate.

9 $${f}_{t}= {\sigma }_{g}\left( {w}_{f }{X}_{t}+ {U}_{f }{h}_{t-1}+{b}_{f}\right)$$

10 $${i}_{t}= \sigma \left( {w}_{i }{X}_{t}+ {U}_{i }{h}_{t-1}+{b}_{i}\right)$$

11 $${C}_{t}{\prime}={\sigma }_{g}\left({w}_{o }{X}_{t}+ {U}_{o }{h}_{t-1}+{b}_{o}\right)$$

12 $${O}_{t}= \sigma \left( {w}_{o }{X}_{t}+ {U}_{o }{h}_{t-1}+{b}_{0}\right)$$

13 $${h}_{{t}_{n}}= {O}_{{t}_{n}}*{h}_{t}\left({c}_{t}\right)$$

14 $${C}_{t}= {F}_{t}{C}_{t-1}*{i}_{t*}{C}_{t}$$

15 $${y}_{n}= \varphi \left({\theta }_{y}{h}_{t}+{b}_{y}\right)$$

where input gate, output gate and forget gate has the following terms $${i}_{{t}_{n}}, {f}_{{t}_{n-1}}, {O}_{{t}_{n}}$$ and $${C}_{{t}_{n}} are the current cell at time t$$, $${h}_{{t}_{n-1}}$$ Is the output value of each cell in the hidden layers. $${h}_{{t}_{n}}$$ Is the value of the hidden layer at time t. $$\sigma$$ is the sigmoid function, $$\varphi$$ is the output of the activation function.

#### Gated Recurrent Unit (GRU)

**Fig. 4:**
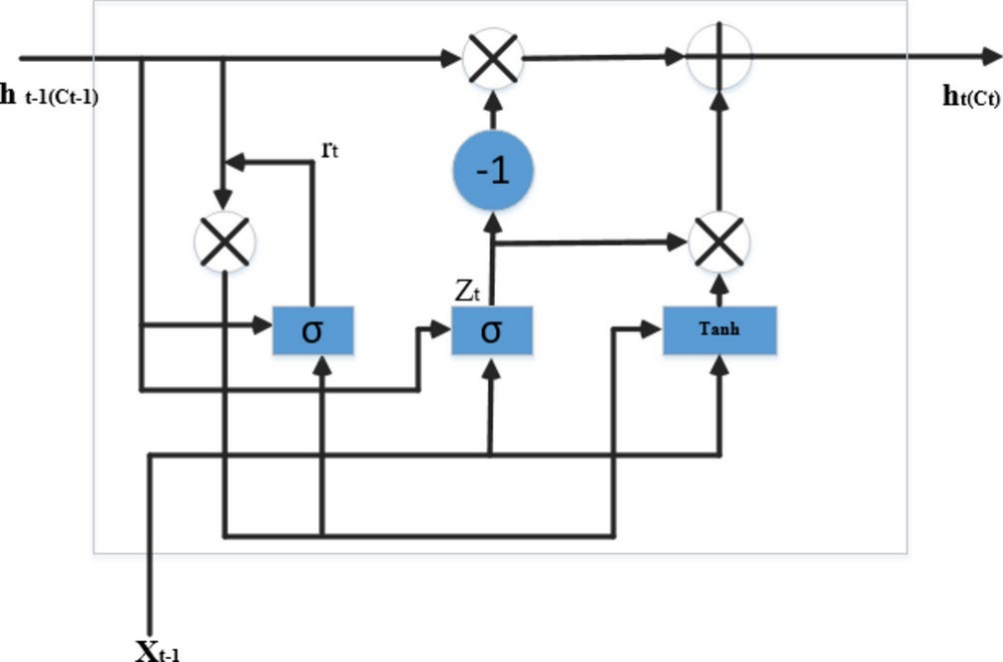
GRU cell structure.

GRU is an improved version of the LSTM and a recurrent neural network. The main difference between LSTM and GRU is that GRU uses fewer hyperparameters than LSTM because it only has two gates, the reset and update gates. The specific architecture of GRU is shown in Fig. 4. The hidden state (hi) and the cell state (ci) merged in GRU. Two control gates include an update gate that controls the amount of information transferred from one layer to the other; the reset gate (ri) specifies the amount of information from the previous layer and is written in the current. The structure of the GRU cell is shown in Fig. 4, GRU equations listed from Eqs. 16 to 20. The reset and update gates are vectors to determine which information should be passed to the output.

16 $${Z}_{t}=\sigma \left({w}_{zh}.{h}_{t-1}+{w}_{zx.}.{x}_{t}+{b}_{z}\right)$$

The input $${x}_{t}$$

17 $${r}_{t}=\sigma \left({w}_{rh}.{h}_{t-1}+{w}_{rx.}.{x}_{t}+{b}_{r}\right)$$

18 $${C}_{t}{\prime}=tanh\left({w}_{xc}.{x}_{t}+{w}_{hc}({r}_{i}* {h}_{t-1})+{b}_{c}\right)$$

19 $${h}_{t}{\prime}=tanh\left({w}_{xh}.{x}_{t}+{r}_{t.}.{w}_{hh}+{h}_{t-1}\right)$$

20 $${h}_{t}={{z}_{t}h}_{t-1} +(1-z){h}_{t}{\prime}$$

where ${w}_{xr}$ , ${w}_{hr}, {w}_{xz}$ Are the weights of the matrices and ${b}_{z}, {b}_{r}$ are the basis vectors. ${z}_{i}, {r}_{i}$ the activation of the update and reset gate.

## Proposed architecture (LSTM_GRU) model

The proposed model utilizes both LSTM and GRU models. The composite LSTM-GRU framework is designed to address specific challenges in predicting transformer health indices that may not be effectively tackled by individual LSTM or GRU models alone. LSTM can excel in capturing long-term dependencies, while GRU can efficiently handle shorter-term dependencies and reduce computational overhead. In addition, utilizing LSTM and GRU in a multi-task learning setup can enhance the model’s learning capacity by enabling it to extract diverse patterns and dependencies from the data for different tasks simultaneously.

**Fig. 5:**
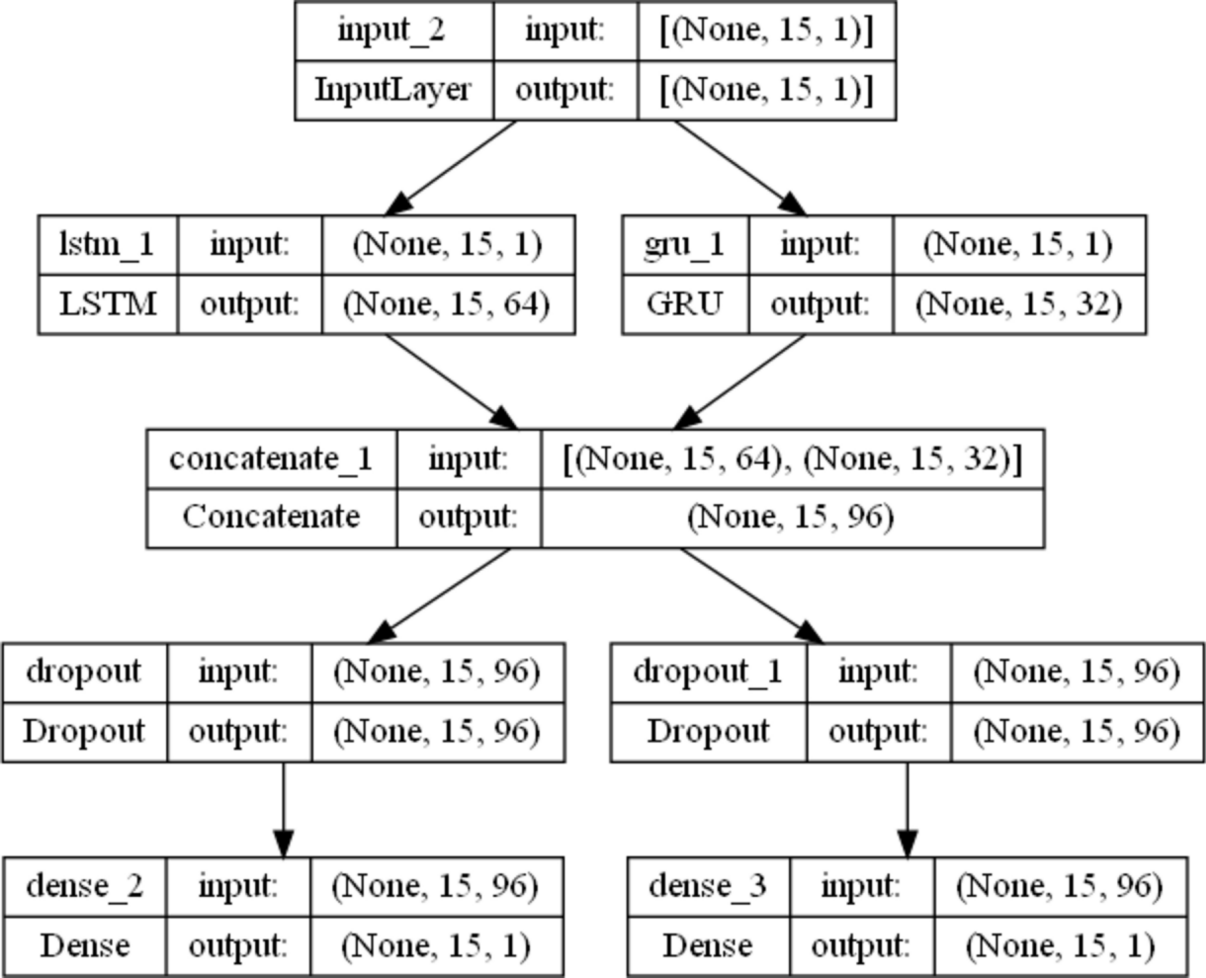
The architecture of the proposed LSTM_GRU Mutlitask model.

The proposed model has a complex architecture, the three gates used to control the flow of information through the cell state. The proposed model was implemented using the Keras library with the TensorFlow backend. Figure 5 shows the architecture of the proposed LSTM_GRU multitask model. The GRU model is also used to control the information flow between layers. The LSTM_GRU layer utilizes both LSTM and GRU layers in the inputs; then, the output is concatenated to capture the dependencies. Dropout was applied to avoid overfitting, followed by a dense layer for each task. The sigmoid activation function was applied for the two regression tasks in addition to mean square error to measure the loss.

Adam optimizer (LR = 0.001) was used in our model. The training was for 500 epochs with 32 batch sizes. Training of the proposed model. Once the feature was extracted from the LSTM and GRU layers, it fed into two dense layers for two regression tasks based on the sigmoid function. To find the best model architecture, after several experiments, we found that utilizing LSTM, GRU with three hidden layers with a multilayer network is the best model in addition. For tuning the model hyperparameters, we used grid search, search for the best LSTM units [32,64,128], best GRU units [16,32,64], and the dropout rate[0.01,0.001,0.001]. We found that utilizing LSTM_layer with 64 neurons, the GRU layer with 32 neurons, and a dropout rate of 0.001 give the best performance. We follow the standard model evaluation technique by dividing the data by 60%, 20%, and 20% for training, validation, and testing, respectively. All results reported on the unseen part of the data. Algorithm 1 shows the overall steps of the proposed model.

### Explanation AI

Although metrics such as accuracy, precision, and recall can offer some insight into the performance of a model, they do not always provide a comprehensive assessment of its reliability. Hence, Explainable AI plays a crucial role in understanding and learning, which entails using tools to provide lucid and understandable explanations for a model’s decisions. This relatively new and sophisticated approach can motivate individuals to deepen their knowledge and understanding of the subject matter. There are various well-known tools available that can provide these explanations, including LIME^28^, SHAP^29^, DeepLIFT^30^, DeepSHAP^31^, etc.

### SHAP

SHAP (Shapley Additive Explanations) is a powerful tool that can generate precise and intelligible interpretations for ML predictions. It offers a unified framework for determining feature importance across an entire dataset and on an individual prediction level. The SHAP library is based on shapely values and game theory, allowing for assigning feature contributions to each variable. Moreover, the SHAP library is versatile and can explain various machine-learning models. It works by constructing an explanation model, called g, for the original model f.

### LIME

LIME (Local Interpretable Model-Agnostic Explanation) is a technique that can be used to interpret ML predictions by approximating a black-box model with a local explanation model. LIME achieves this by fitting and explaining a local model per instance. The interpretable inputs, along with the mapping X = h_x(X^'), are used to convert the binary vector of the interpretable input into the original input.

## Results and discussion

This section details the results of all experiments. In this study, the results are divided into three main stages. The first stage used ML models, including traditional and ensemble ML models in both prediction tasks; the second used traditional and hybrid DL models for each task. The third is to use multitask DL to predict the two tasks concurrently. All models were evaluated using various evaluation metrics that are detailed in Sect. "SHAP".

**Algorithm 1:**
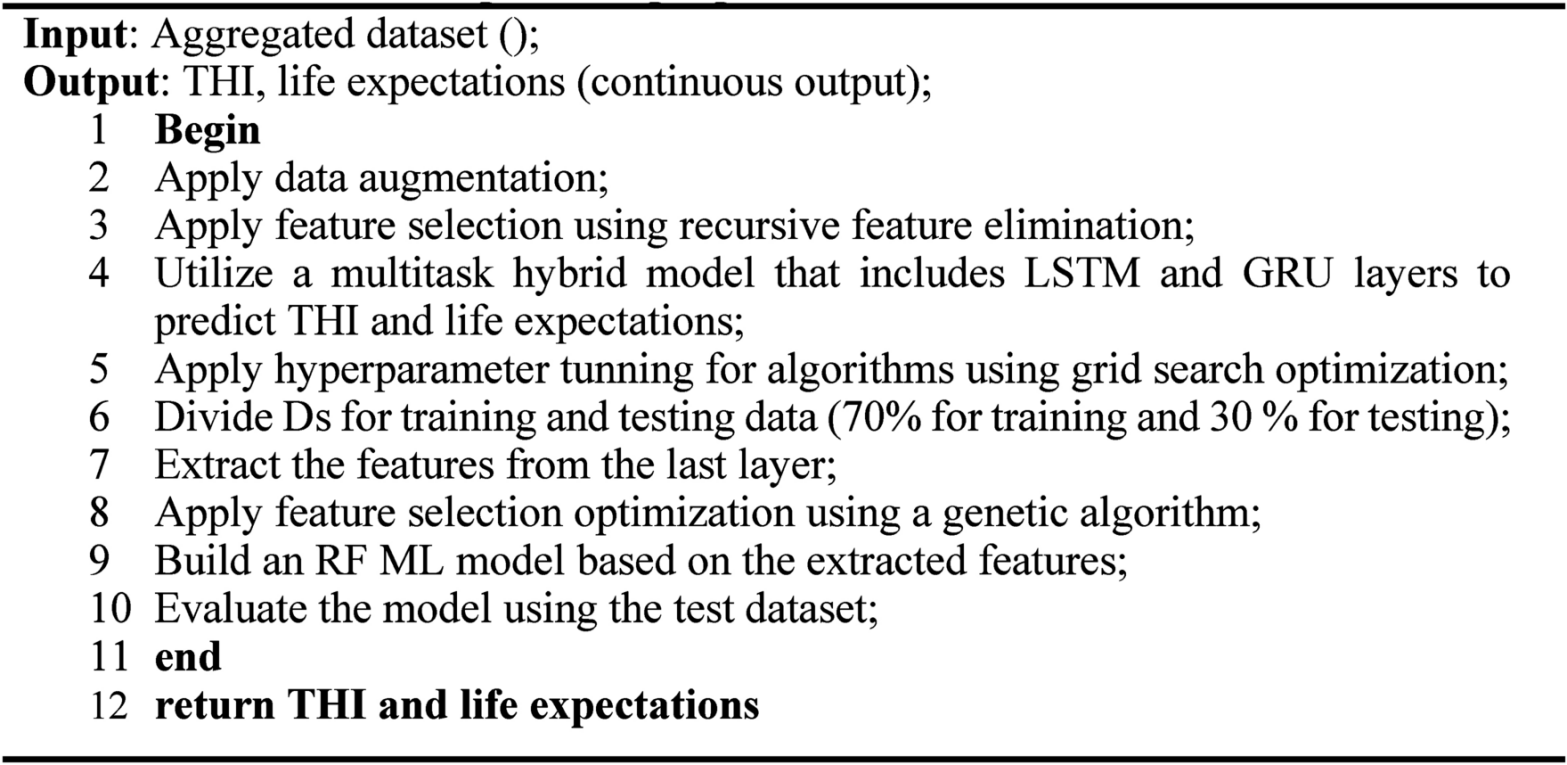
Overall steps of the proposed model.

### Experimental setting

All results are on an NVIDIA server, intelcorei5 CPU with 16 GB RAM. This ensured that the proposed framework could effectively work in real applications. Our research was done on Python 3.9. Al preprocessing stages were conducted using NumPy, pandas, and Scikit-learn libraries. All DL models are built using the Keras library with TensorFlow backend. This study utilized several ML all including LR, RG, DT, RF, and Gradient Boosting. In addition, several DL models include LSTM, CNN, and GRU. All models are optimized using grid search. Table 6 shows all ML algorithms, and Table 9 shows hyperparameters for the DL model.

**Table 6:** ML parameters for utilized ML.

Model	Hyperparameters for ML models
LR	fit_intercept = True, normalize = True,copy_X = True, n_jobs = − 1
Ridge	Alpha = 0.8
RF	n_estimators = 100, max_depth = 20, random_state = 33
DT	max_depth = 12, random_state = 33
Gradient boosting	n_estimators = 100, max_depth = 30, learning_rate = 1.5, random_state = 33

### Evaluation metrics

To evaluate the performance of the developed model, several models were utilized, including Mean Absolute Error, Median Absolute Error (MedAE), (MAE), Mean Square Error (MSE), and R^2^ score. These metrics are computed as follows:

13 $$MAE=\frac{1}{n} {\sum }_{i=1}^{n}\left|{y}_{i}-{\widehat{y}}_{i}\right|$$

14 $$MSE=\frac{1}{n} {\sum }_{i=1}^{n}{\left({y}_{i}-{\widehat{y}}_{i}\right)}^{2}$$

15 $$MedAE=median(\left|{y}_{1}-{\widehat{y}}_{1}\right|, \left|{y}_{2}-{\widehat{y}}_{2}\right|, \dots , \left|{y}_{n}-{\widehat{y}}_{n}\right|)$$

16 $${R}^{2}=1- \frac{{\sum }_{i=1}^{n}{\left({y}_{i}-{\widehat{y}}_{i}\right)}^{2}}{{\sum }_{i=1}^{n}{\left({y}_{i}-\underline{y}\right)}^{2}}$$

where ${y}_{i}$ denotes the ith true value, ${\widehat{y}}_{i}$ denotes predicted value, $n$ denotes the number of instances and $\underline{y}$ denotes true values.

### First machine learning model (THI)

This section explores the role of ML in life expectations and THI and life excepectations in a single-task model. Firstly, we utilize ML to predict the transformer’s health index, which considers crucial technical information, and help ensure the transformer’s reliability, safety, and longevity. We conducted 20 experiments in this to find the best ML model that could be utilized to give the most accurate prediction. We plan first to test all features with the default parameters, then choose the best feature using feature optimization, optimize the classifier hyperparameters, then combine the best features with the optimized hyperparameter.

**Fig. 6:**
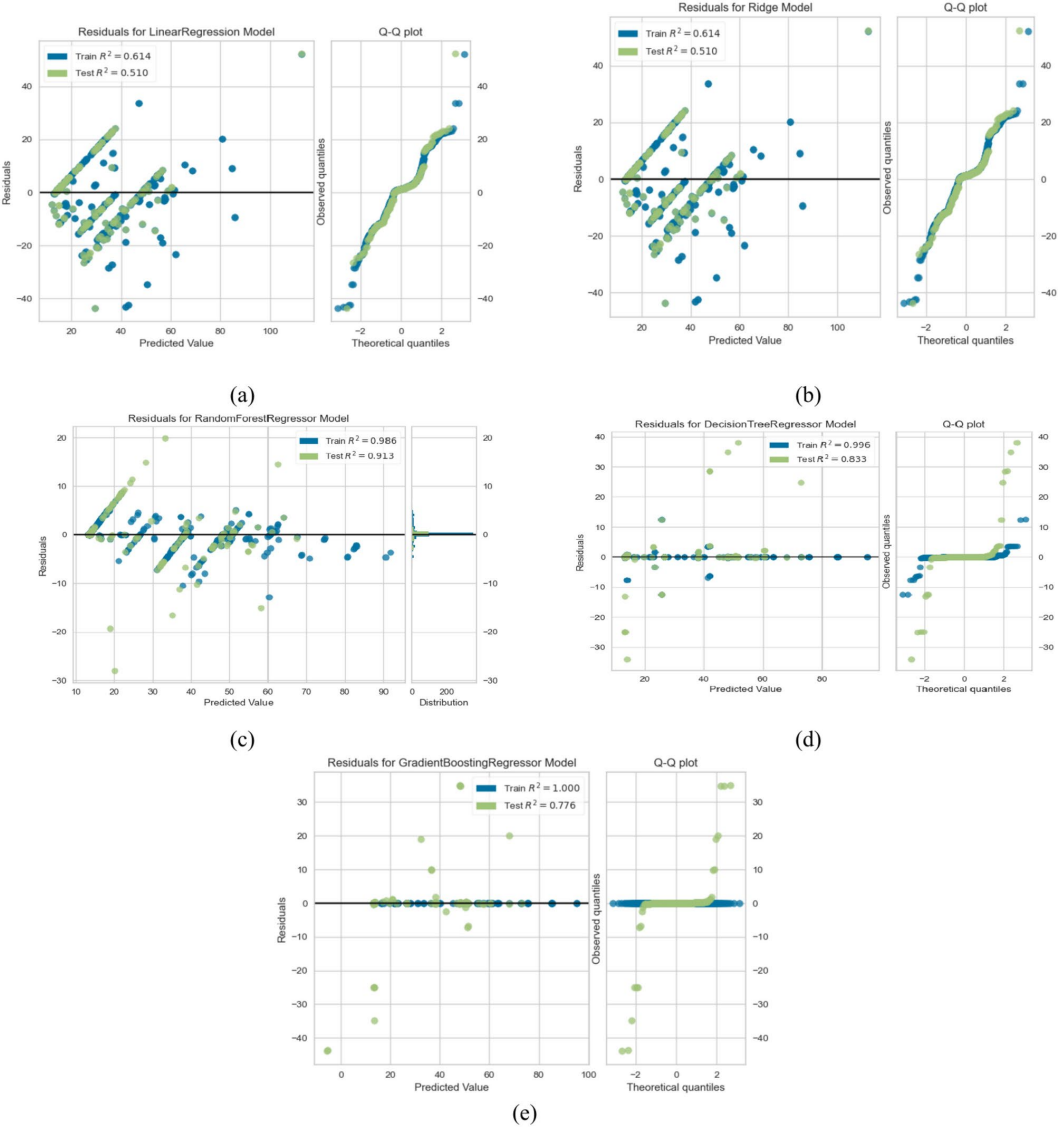
QQ plot for ML algorithms for THI task. (**a**) linear regression (**b**) rdge ression (**c**) Random Forest (**d**) decision tree (**e**) gradient boosting.

Table 7 shows the training and testing scores of the utilized models and the other evaluation metrics, including MAE, MSE, medAE, and the r2 score. From Table 7, we can observe the following (1) Utilizing the traditional ML without FS or hyperparameter optimization gives the least performance in all regression algorithms (2). As expected, optimizing the hyperparameters using grid search optimization improves the overall performance of some algorithms. LR and Ridge’s regression gives the least training and testing scores, 0.613 and 0.512, respectively. In addition, it achieved the worst performance in terms of the other evaluation metrics MAE = 134.95, MSE = 8.23, MedSE = 4.890, and R2 score = 0.511; This performance was not much changed when applying feature selection on these algorithms. It improved by about 0.1–0.3 for most evaluation metrics (3). The tree-based algorithms include DT and ensemble tree-based algorithms include RF and gradient boosting to improve the overall performance (3). applying both hyperparameter optimization and feature selection, the best performance obtained from RF with 100 estimators and 12 depth that utilized to improve the over MAE = 23.917, MSE = 2.64, MedSE = 0.438, and R2 score = 0.913. Figure 6a to e show the Q–Q plot for the five algorithms (in the case of combined hyperparameter optimization and feature selection). From that curve, we can observe that DT and RF, which give the best performance, roughly follow the straight line, indicating that the residual is normally distributed. Where the QQ plot of the linear ridge model deviates from the straight line, which assures that not normally distributed and it needs for alternative to ensure fair comparison, Fig. 8a show comparison between all algorithms in terms of statistical test, and Fig. 9a shows the feature importance of all features in predicting THI.

**Table 7:** The results of ML in predicting THI.

Model	Train score	Test score	MSE	MAE	MedAE	R2 Score
Base results						
LR	0.521	0.501	149.71	10.322	6.299	0.489
Ridge	0.521	0.501	149.71	10.322	6.299	0.489
RF	0.921	0.882	27.716	3.936	2.832	0.802
DT	0.901	0.801	50.03	3.891	2.432	0.809
GB	0.931	0.610	66.15	4.167	1.231	0.690
Results with hyperparameter optimization						
LR	0.541	0.500	147.91	9.322	5.387	0.499
Ridge	0.541	0.500	147.91	9.22	5.387	0.499
RF	0.961	0.891	27.716	3.566	1.738	0.811
DT	0.921	0.811	50.03	2.771	1.836	0.821
GB	0.931	0.625	66.15	3.119	1.031	0.710
Results with feature selection						
LR	0.571	0.510	144.82	9.322	5.196	0.5001
Ridge	0.571	0.500	144.85	9.22	5.196	0.5101
RF	0.922	0.902	25.726	3.566	1.638	0.8323
DT	0.955	0.821	48.03	2.771	1.376	0.8220
GB	0.982	0.725	62.15	3.119	0.291	0.7415
Results with feature selection & hyperparameter optimization						
LR	0.613	0.510	134.95	8.222	4.896	0.5101
Ridge	0.613	0.510	134.95	8.222	4.896	0.5101
RF	0.9859	0.9131	23.917	2.466	1.438	0.913
DT	0.955	0.8330	46.01	1.872	0.2761	0.8330
GB	0.982	0.775	61.85	2.09	0.0361	0.775

### First machine-learning model (life expectation)

Predicting the transformer’s life expectancy post-THI analysis is necessary for maintenance, cost planning, and system reliability. Twenty experiments were conducted to predict the life expectancy of the transformer. They were categorized as follows: the first group with five experiments based on ML algorithms, another group based on algorithms that optimized with grid search, five with features chosen with feature selection optimization, and the last group with five experiments that combined the optimized features and the optimized hyperparameters.

**Fig. 7:**
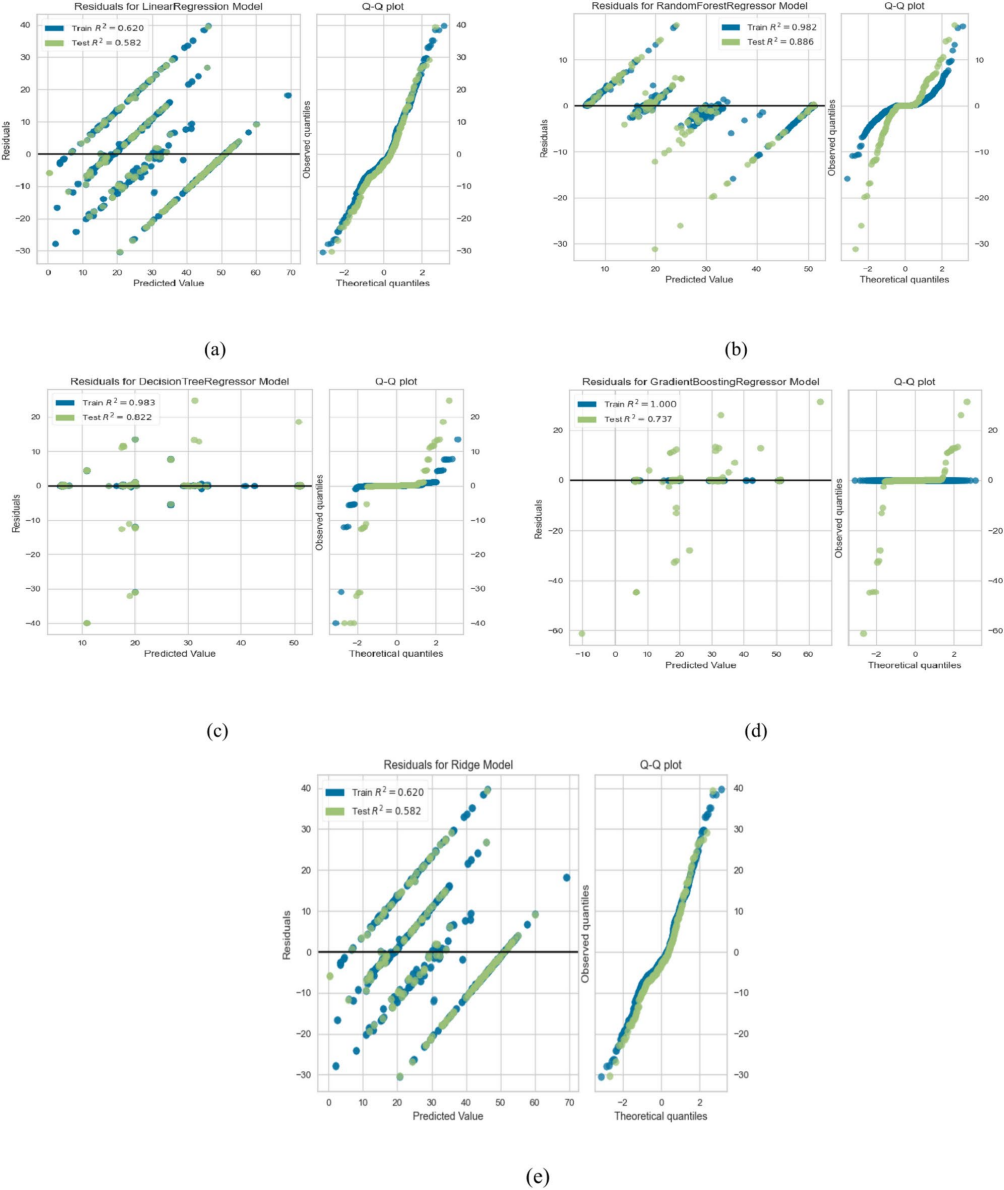
QQ plot for ML algorithms for timelife exception task. (**a**) linear regression (**b**) rdge ression (**c**) Random Forest (**d**) decsion tree (**e**) gradient boosting.

**Fig. 8:**
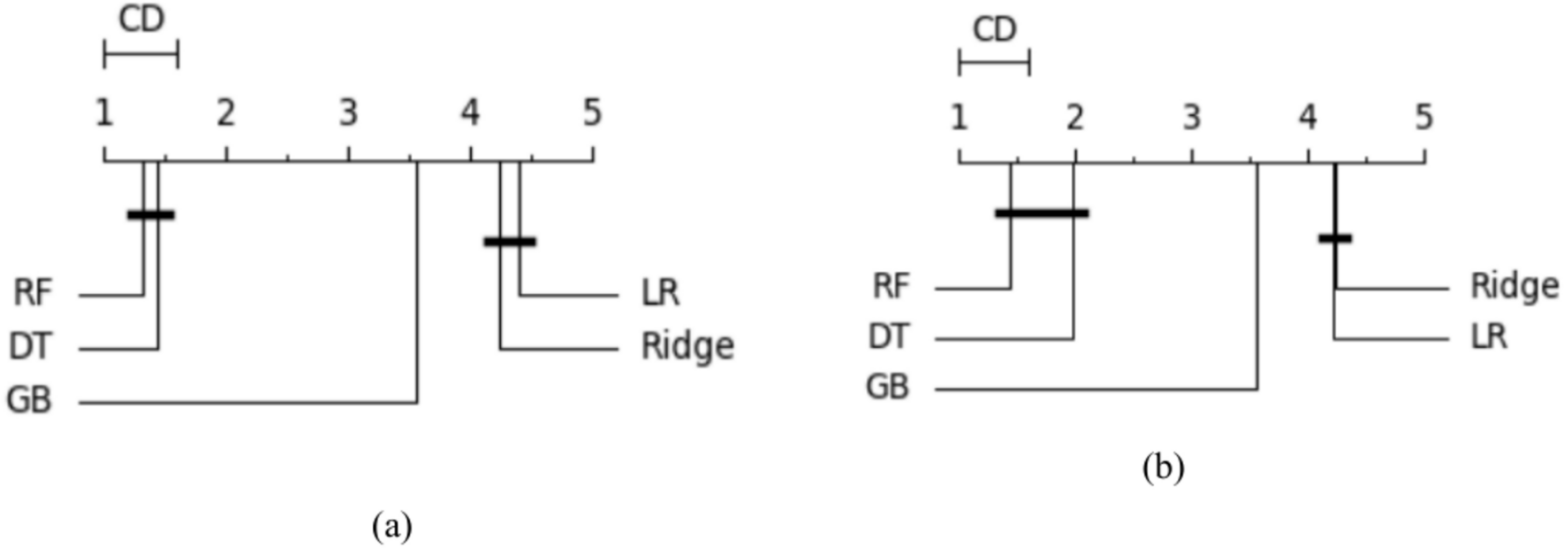
Statistical comparison between all ML algorithms (**a**) comparison between algorithms in predicting THI task (**b**) comparison between algorithms in predicting time life expectation task.

**Fig. 9:**
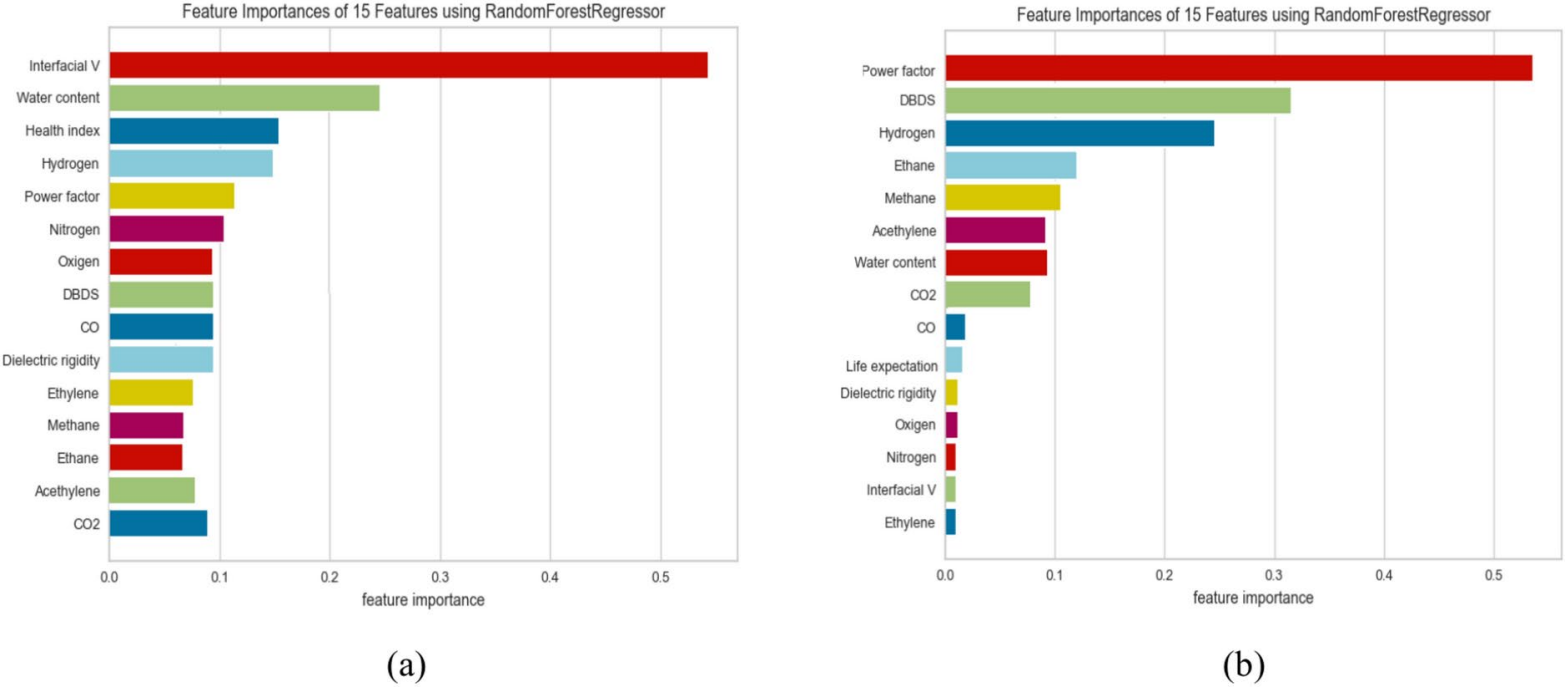
Feature importance in predicting THI and timelife expectation (**a**) THI, (**b**) timelife expectations.

As shown in Table 8, the training and testing scores of the utilized models and the other evaluation metrics, including MAE, MSE, medAE, and the r2 core are depicted. We make the following observations: (1) LR and Ridge regression give the least training and testing scores, 0.620 and 0.582, respectively, in the base results. And MAE = 133.49, MSE = 8.92, MedSE = 6.72, and R2 score = 0.582. (2) This result improved when optimizing the hyperparameters with grid search for the tree-based algorithms, including the single tree-based (DT) and ensemble tree-based algorithms (RF and gradient boosting). (3) when applying feature selection optimization, the overall performance improved for some algorithms, including GB, RF, and DT (4) The best performance obtained from RF was achieved with max depth = 16 and 200 estimators (MSE = 36.43, MAE = 3.392, MedSE = 1.082, and R2 score = 0.885). Figure 7a to e show the Q-Q plot for the five algorithms. From that curve, we can observe that DT and RF, which give the best performance, roughly follow the straight line, indicating that the residual is normally distributed. The QQ plot of the linear ridge model deviates from the straight line, which assures that it is not normally distributed and needs an alternative. To compare the performance of all ML algorithms from a statistical point of view, Fig. 8a, b shows the comparison between ML algorithms in terms of the Friedman and Nemnyi test. It shows that Rf outperforms other ML algorithms. Figure 9a, b shows the feature importance of all features in predicting life exceptions (Table 9).

**Table 8:** The results of ML in predicting timelife expectations.

Model	Train score	Test score	MSE	MAE	MedAE	R2 score
Base results						
LR	0.560	0.522	145.11	10.11	8.43	0.511
Ridge	0.560	0.532	145.11	10.11	8.43	0.511
RF	0.781	0.655	59.43	4.89	1.882	0.885
DT	0.732	0.621	60.03	3.99	1.765	0.821
GB	0.791	0.736	89.43	4.112	1.981	0.736
Results with alogthims that optimized with grid search						
LR	0.572	0.531	143.11	9.810	7.43	0.521
Ridge	0.572	0.531	143.11	9.810	7.43	0.521
RF	0.791	0.525	58.43	4.892	1.882	0.765
DT	0.762	0.691	58.92	3.921	1.209	0.701
GB	0.791	0.736	88.71	3.656	1.007	.656
Results with feature selection optimization						
LR	0.620	0.582	133.49	8.921	6.72	0.576
Ridge	0.620	0.582	133.49	8.921	6.72	0.562
RF	0.931	0.835	39.43	3.392	1.179	0.725
DT	0.923	0.801	58.03	2.462	0.179	0.711
GB	1	0.736	86.19	2.656	0.551	0.721
Results with feature selection & hyperparameter optimization						
LR	0.620	0.592	122.49	8.063	5.72	0.582
Ridge	0.620	0.592	122.49	8.063	5.72	0.582
RF	0.981	0.885	36.43	3.192	1.082	0.885
DT	0.983	0.821	57.03	2.22	0.079	0.821
GB	1	0.736	84.11	2.600	0.081	0.736

**Table 9:** Hyperparameter of developed DL models.

Models	Model Hyperparameters and values
LSTM	Input layer = 37, Number of layers = 6, Regularization (L2 = 0.1), Batch size = 32, Number of epochs = 10, Dropout = 0.1 Activation function in hidden layers = ReLU, Optimizer = ADAM
CNN	Desne_units = 128, Dropout rate: 0.3, convolutional filters: 64, Kernel size = 3 × 3, max pooling_size = 2 × 2
GRU	Dense_units = 256, Dropout rate = 0.2, GRU_units = 128, ‘optimizer’: ‘rmsprop’
LSTM-CNN	Dropout_rate’: 0.2, ‘gru_units’: 32, ‘lstm_units’: 128, ‘optimizer’: ‘rmsprop’}
LSTM- GRU	Dense_units = 128, Dropout_rate = 0.2, filters = 128, gru_units = 128, kernel_size = 5, 'optimizer = rmsprop
CNN-GRU	Dense_units = 256, second_dense = 128, Dropout rate = 0.3, Number of GRU units: 128, Number of CNN filters = 64, Kernel size for CNN layers: 5 × 5, Pooling size for max pooling layers: 2 × 2, Number of dense layers: 2, Optimizer = Adam

### Single-task deep learning for predicting THI and timelife expectations

In this section, we explore the role of DL in predicting THI of the transformer. Three DL models include LSTM, CNN, and GRU, and three hybrid DL models, LSTM-CNN, LSTM, GRU, and CNN_GRU models, were utilized for prediction.

### Single deep learning model THI (Single task)

**Fig. 10:**
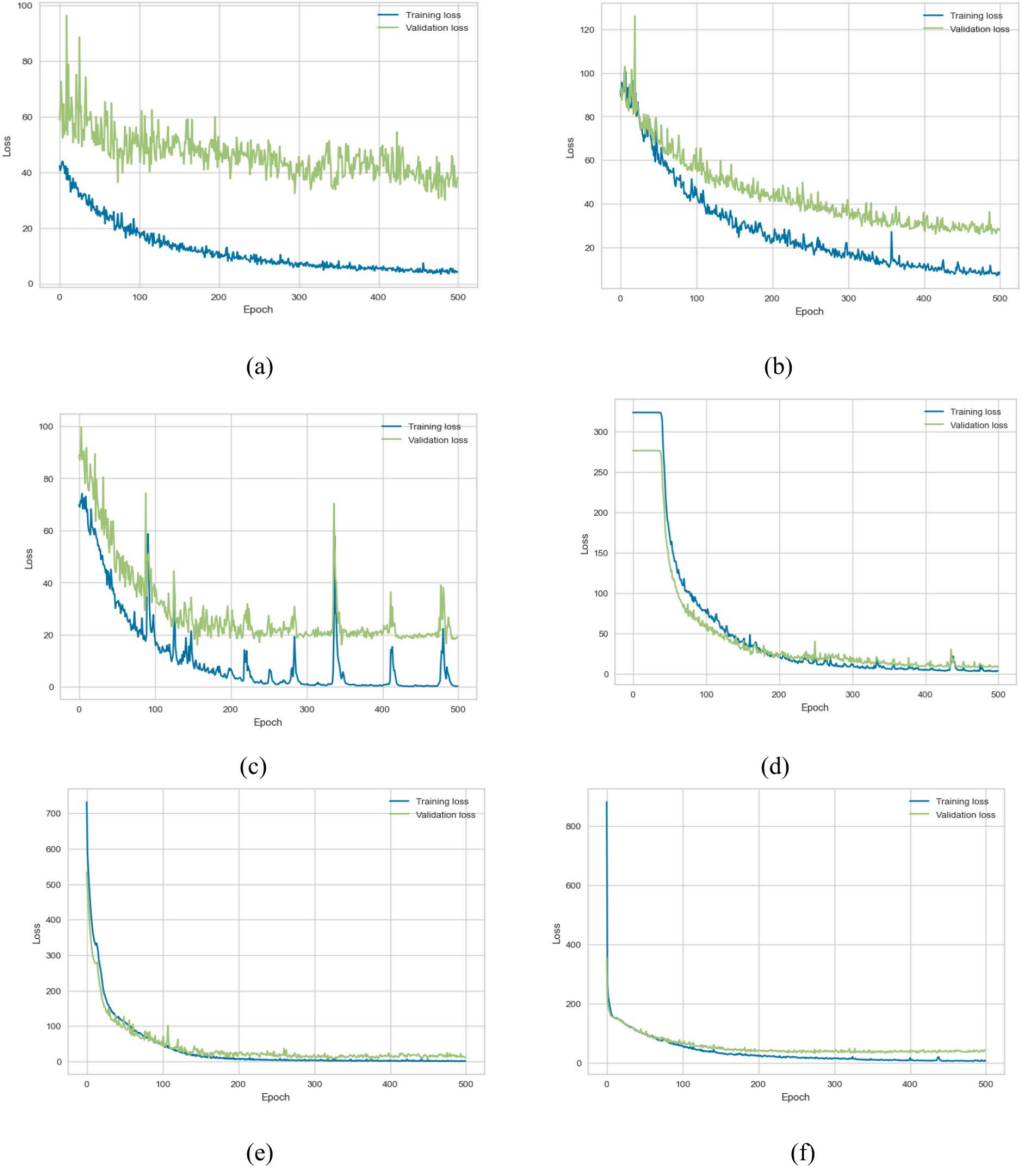
Validation curve for predicting THI for all utilized algorithms.

In this section, we explore the role of DL models in predicting THI of the transformer by using feature selection optimization and hyperparameter tuning, and we conducted 30 experiments to reach the optimum regressor. Table 10 includes evaluation measures for all models with various metrics. We make the following observations: (1) utilizing the DL model improves the overall performance in prediction, R2 score improved by about 2–10% for all utilized DL models. (2) GRU gives the best performance of the DL (MSE = 27.821, MAE = 2.41, MedSE = 2.004, r2 score = 0.899) (3) The overall performance improved with hybrid DL models. This return for several reasons, including increased generalization ability and flexibility of the hybrid DL model to the single DL model. LSTM gives the best performance; it achieves a promising performance of (MSE = 11.442, MAE = 1.28, MedSE = 0.821, r2 score = 0.958). (3) Fig. 10a to f show the loss of the model both in terms of training and validation data. From that visualization, we could assure that the LSTM_GRU model gives the best performance as it gives more stable data in the prediction.

**Table 10:** The results of DL model for predicting THI.

Model	MSE	MAE	MedSE	R2 Score
Base results				
LSTM	42.324	3.650	3.432	0.792
CNN	40.170	3.471	3.591	0.786
GRU	35.621	3.381	3.224	0.789
LSTM-GRU	17.762	2.628	1.861	0.863
CNN-GRU	39.742	2.815	1.843	0.821
LSTM-CNN	26.650	2.227	1.772	0.892
Agorthims with hyperparameter optimization				
LSTM	40.214	3.150	2.952	0.801
CNN	39.080	3.961	2.972	0.826
GRU	31.621	3.113	3.004	0.849
LSTM-GRU	16.142	2.323	1.341	0.891
CNN-GRU	36.431	2.625	1.652	0.865
LSTM-CNN	23.220	1.977	1.031	0.899
Results with feature selection optimization				
LSTM	39.91s9	2.860	2.663	0.811
CNN	39.190	2.971	2.611	0.836
GRU	27.821	2.991	2.244	0.841
LSTM-GRU	15.460	1.28	1.001	0.908
CNN-GRU	35.644	1.935	1.083	0.878
LSTM-CNN	21.20	1.47	0.962	0.902
Results with feature selection & hyperparameter optimization				
LSTM	38.24	2.250	2.132	0.8611
CNN	39.490	2.471	2.031	0.8566
GRU	27.821	2.41	2.004	0.899
LSTM-GRU	11.442	1.28	0.821	0.958
CNN-GRU	33.554	1.935	0.883	0.888
LSTM-CNN	19.20	1.47	0.632	0.939

### Deep learning (Single task-timelife expectation)

**Fig. 11:**
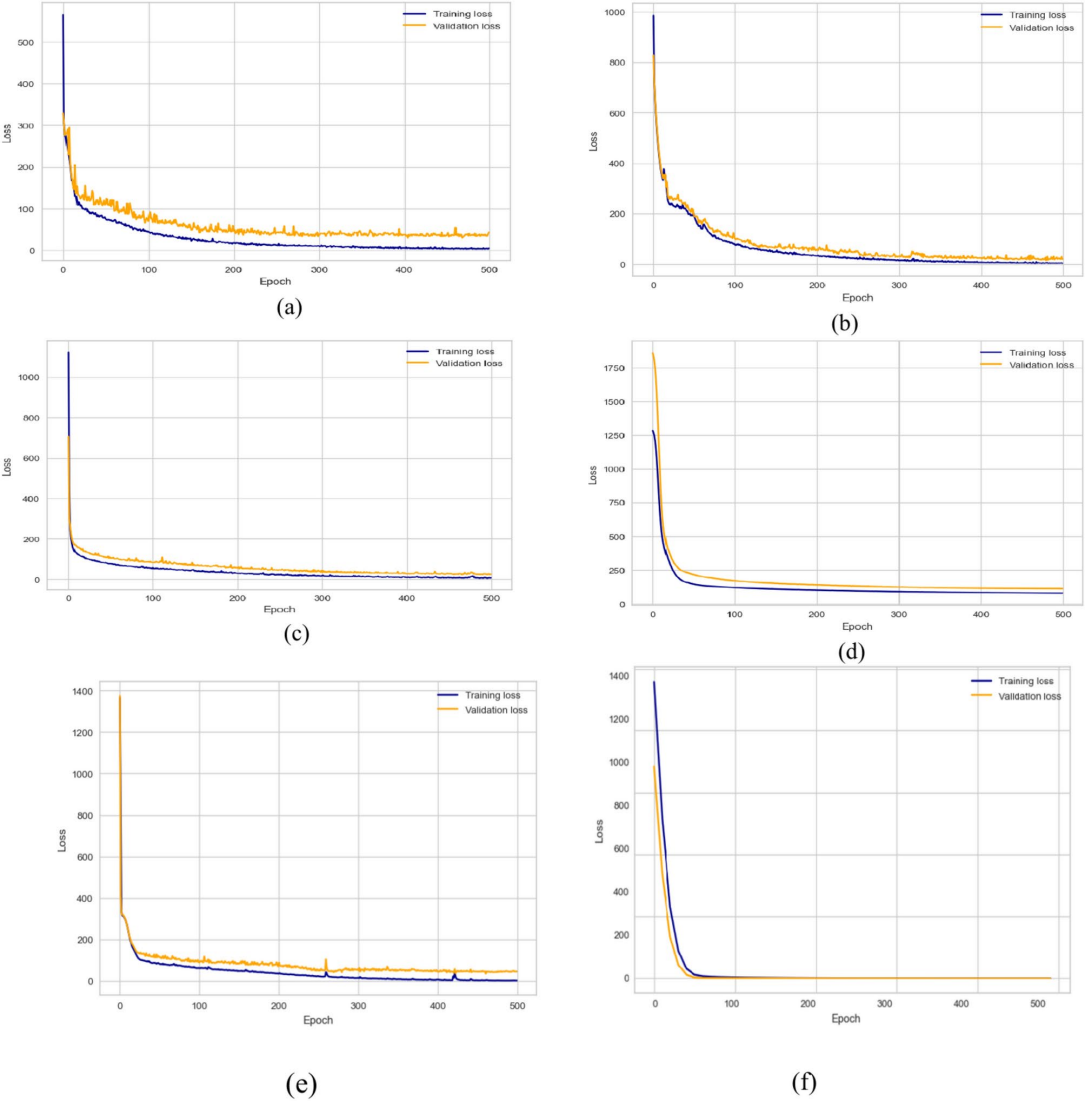
Validation curve for all single-task DL models for predicting life expectations for all utilized algorithms.

**Fig. 12:**
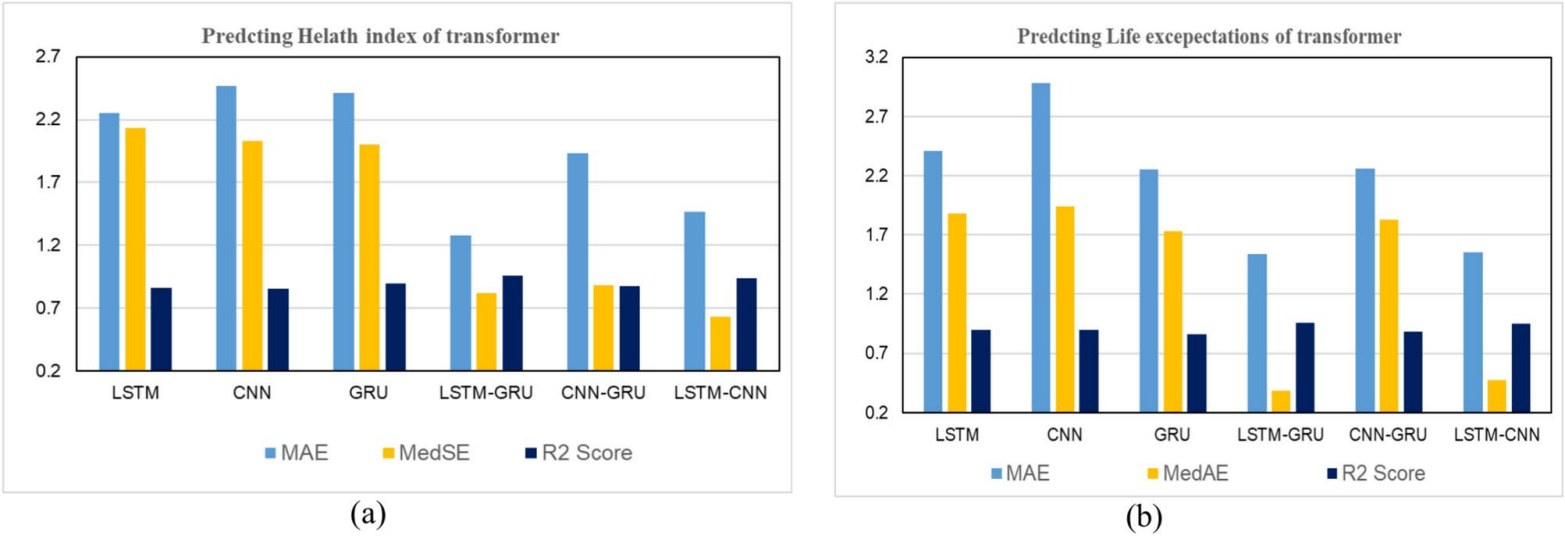
Comparison between all DL models (single task) (**a**) for THI task, (**b**) timelife expectation task.

**Fig. 13:**
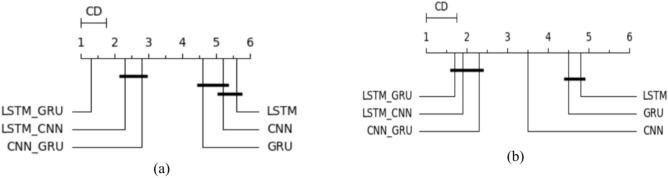
A statistical comparison between all single task DL algorithms (**a**) comparison between algorithms in predicting THI task (**b**) comparison between algorithms in predicting timelife expectation task.

In this section, we explore the role of DL in predicting the life expectation of the transformer. First, we explore the role of LSTM, CNN, and GRU, in addition to three hybrid DL including LSTM-CNN, LSTM, GRU, and CNN_GRU. Table 11 includes evaluation measures for all models with various metrics. We make the following observations: (1) DL improved the overall performance in prediction, R2 score improved by about 2–4% for all utilized DL models. (2) CNN gives the best performance of the DL (MSE = 29.12, MAE = 2.98, MedSE = 1.943, r2 score = 0.898) (3) Utilizing the hybrid model improves the overall performance, which outperforms all ML and single DL model. It achieves the promising performance of c). (3) Fig. 11a to f show the loss of the model both in terms of training and validation data. Figure 11d shows that the LSTM_GRU model performs best regarding stability and evaluation metrics. Figure 12 shows the comparison between all DL models (single task) (a) for HI task, (b) life exception task. Figure 13 depicts statistical comparison between all single task DL algorithms.

**Table 11:** Results of the DL model for predicting life expectations.

Model	MSE	MAE	MedAE	R2 score
Base results				
LSTM	29.8211	3.612	2.652	0.799
CNN	30.12	2.98	2.654	0.778
GRU	39.246	2.250	2.335	0.781
LSTM-GRU	17.332	2.639	1.254	0.846
CNN-GRU	39.653	2.970	2.831	0.821
LSTM-CNN	19.135	2.633	1.335	0.869
Results with hyperparameter tunning				
LSTM	28.8211	3.121	2.252	0.829
CNN	31.110	3.98	2.421	0.828
GRU	37.142	2.460	2.311	0.831
LSTM-GRU	16.312	2.549	1.032	0.873
CNN-GRU	38.112	2.730	2.291	0.861
LSTM-CNN	18.255	2.443	1.255	0.879
Results with feature selection				
LSTM	27.032	2.931	2.312	0.842
CNN	30.541	3.001	2.231	0.864
GRU	37.142	2.430	2.011	0.853
LSTM-GRU	14.112	2.019	0.8321	0.921
CNN-GRU	38.031	2.620	2.221	0.877
LSTM-CNN	18.255	2.443	1.255	0.916
Results with hyperparameter tuning & feature selection				
LSTM	27.8211	2.412	1.883	0.899
CNN	29.12	2.98	1.943	0.898
GRU	36.246	2.250	1.731	0.861
LSTM-GRU	13.209	1.539	0.387	0.958
CNN-GRU	37.4511	2.260	1.831	0.882
LSTM-CNN	16.157	1.553	0.471	0.949

### Deep learning (Multitask)

**Fig. 14:**
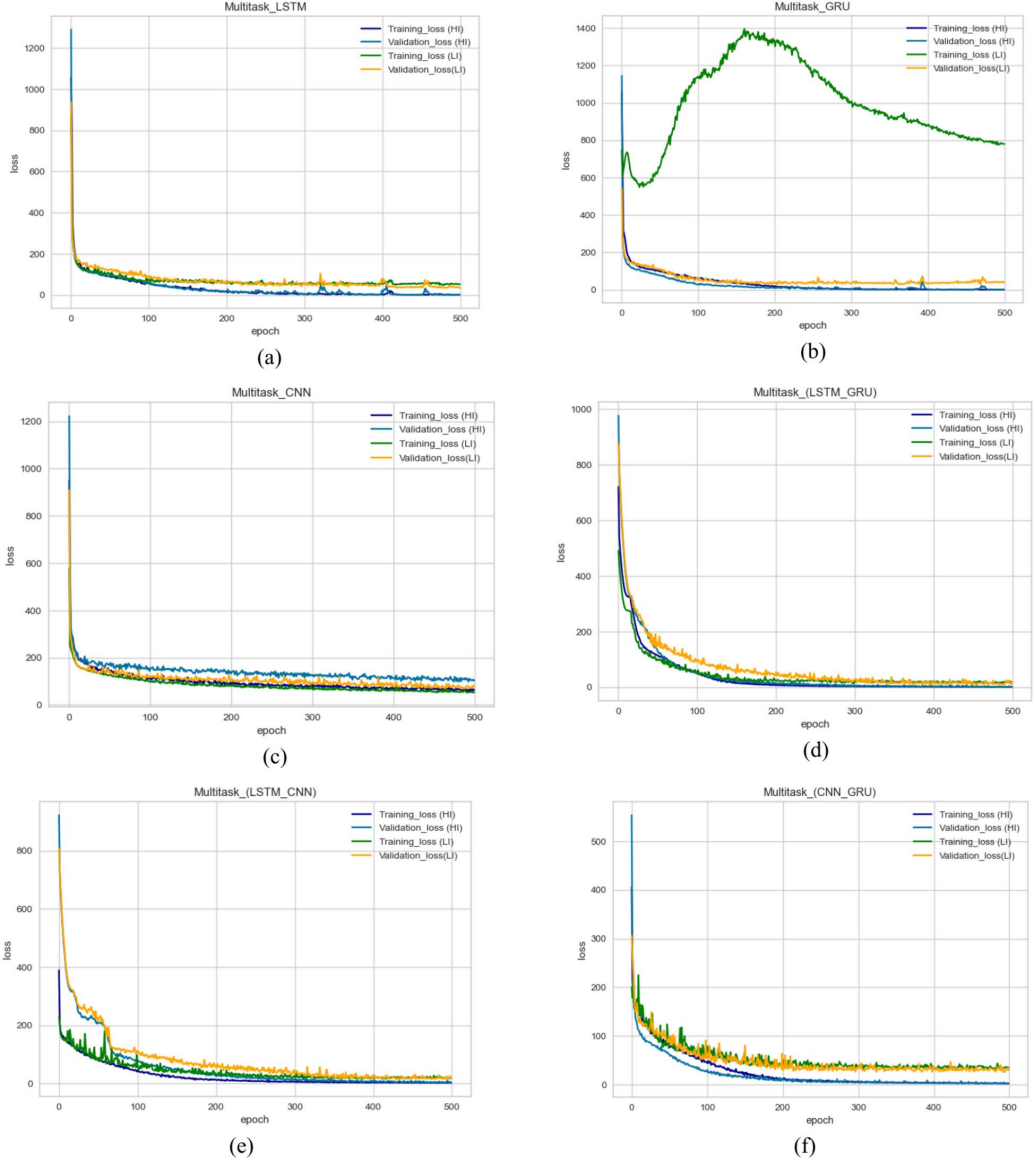
Validation curve for all multitask DL models for predicting both timelife expectations and health index.

**Fig. 15:**
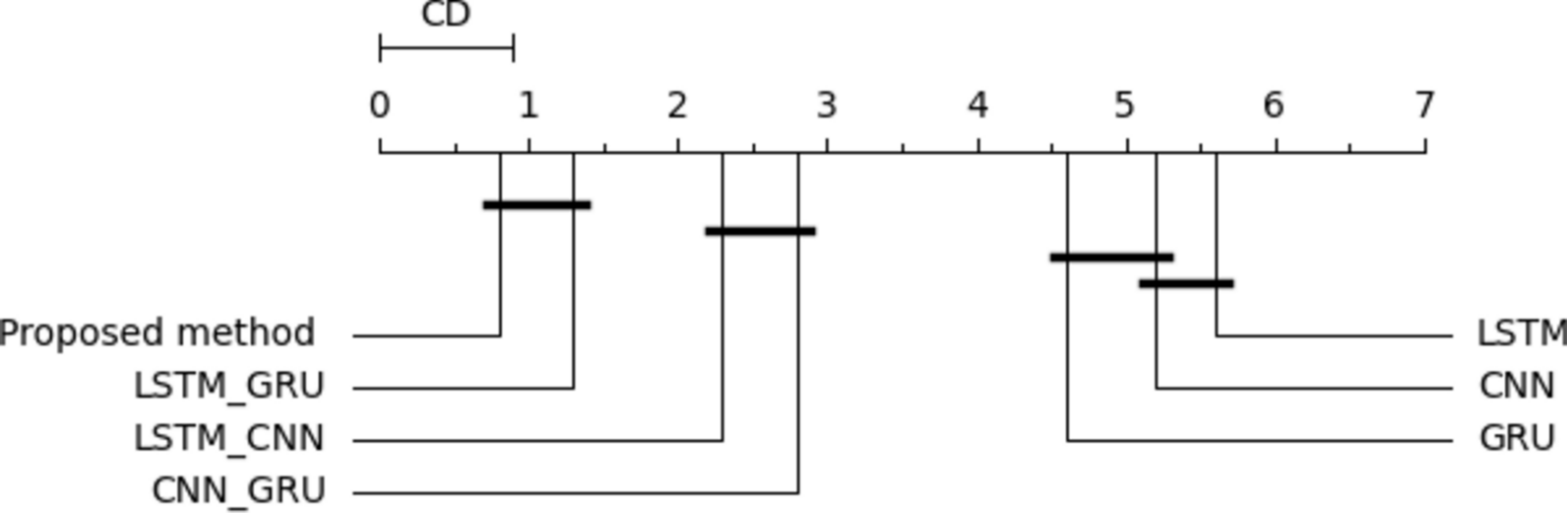
Statistical comparison between multitasking DL Models.

**Fig. 16:**
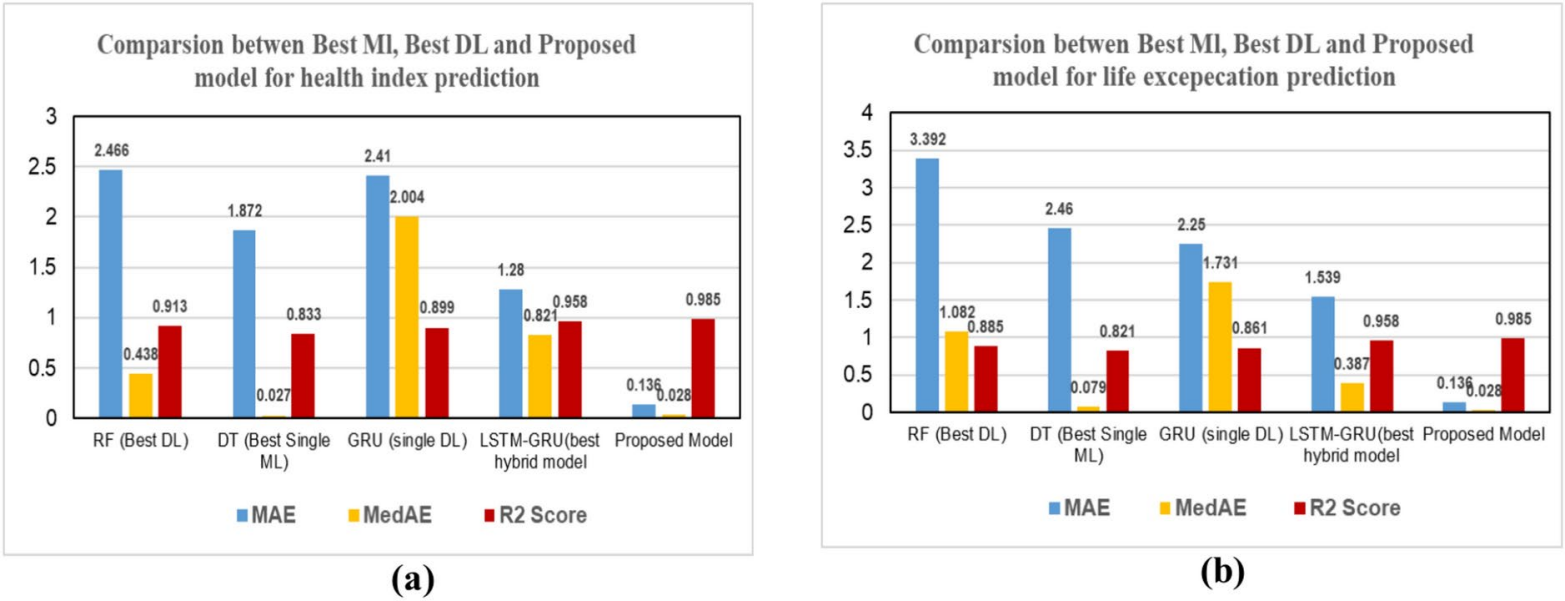
Comparison between all single-task DL models (**a**) THI (**b**) timelife expectations.

Multitask model considers the multi-objective problem where each task works as a regularization for the other task. In this section, we concurrently optimize the two regression tasks. To our knowledge, we are the first study that concurrently predicts both HI and life exception. The final model could tell the engineer the transformer details, which helps make the right decision. We follow the same setting as all previous experiments. In this experiment, we performed the multitask model using single DL and hybrid DL models. As shown in Table 12, utilizing traditional, the best performance was obtained from the GRU model (MSE = 18.162, MAE = 3.583, MedSE = 3.871, r2 score = 0.894). Following that experiment, we tried the same models with optimizing the models based on gird search, optimizing the parameters to enhance the overall performance. The best performance was obtained from LSTM_GRU (MSE = 20.123, MAE = 2.21, MedSE = 2.632, r2 score = 0.911). In the next step, we utilize the selected features to improve the performance of single and hybrid DL models. Combine feature selection and hyperparameter to improve the overall performance. LSTM_GRU model gives the best performance MSE = 18.923, MAE = 1.059, MedSE = 0.2399, r2 score = 0.931. Add an optimization layer over the features extracted from the DL model. We utilized GA to choose the optimum subset after extracting features from the dense layers. Then the chosen features are used to build the ML model over that feature. That model achieves superior performance overall single and hybrid models (MSE = 2.543, MAE = 0.1364, MedSE = 0.0284, r2 score = 0.985). Figure 14 shows the validation curve for all multitask DL models for predicting both life expectations and health index. Figure 15 show the statistical comparison between multitasking DLmodels. To compare between all utilized models (Fig. 16).

**Table 12:** Results of multitask DL model for predicting both THI and timelife expectation.

Model	MSE	MAE	MedAE	R2 Score
Base results				
LSTM	19.87	4.976	3.981	0.90
CNN	26.622	4.887	3.986	0.803
GRU	18.162	3.583	3.871	0.894
LSTM-GRU	21.223	3.611	2.882	0.872
CNN-GRU	36.244	3.321	2.892	0.869
LSTM-CNN	27.612	3.823	2.654	0.880
Proposed model	7.711	2.101	2.321	0.907
Results with hyperparameter optimization				
LSTM	18.27	3.776	2.861	0.92
CNN	25.594	4.987	2.866	0.813
GRU	11.662	3.543	2.431	0.934
LSTM-GRU	20.123	2.221	2.631	0.911
CNN-GRU	33.244	2.43	1.921	0.879
LSTM-CNN	23.692	2.633	1.966	0.901
Proposed model	6.643	2.421	1.973	0.929
Results with feature selection				
LSTM	16.17	3.458	1.961	0.93
CNN	24.494	4.326	1.976	0.833
GRU	10.563	2.186	1.231	0.9521
LSTM-GRU	19.923	2.059	1.231	0.921
CNN-GRU	30.544	2.018	1.321	0.899
LSTM-CNN	20.592	2.422	1.432	0.931
Proposed model	4.543	1.221	1.113	0.922
Results with hyperparameter optimization & feature selection				
LSTM	14.17	2.258	1.263	0.947
CNN	22.494	3.326	1.5729	0.845
GRU	9.563	1.686	0.852	0.965
LSTM-GRU	18.923	1.059	0.2399	0.931
CNN-GRU	29.544	1.818	0.4316	0.907
LSTM-CNN	17.592	1.3023	0.236	0.944
Proposed model	2.543	0.13646	0.0284	0.985

The reliability of the model was demonstrated through a comprehensive evaluation process that involved various performance metrics and statistical analyses. Metrics such as (MSE), (MAE), (MedAE), and the R-squared (R^2^) score were used to assess the accuracy of the model. Additionally, statistical tests, including the Wilcoxon signed-rank test and the Friedman test, were conducted to compare the performance of the model with other algorithms, confirming that the observed differences were statistically significant. The robustness of the model was further validated through the use of explainable AI tools such as SHAP, which provided both global and instance-level insights into the features that significantly influenced the model’s predictions.

## Explanation of proposed model

**Fig. 17:**
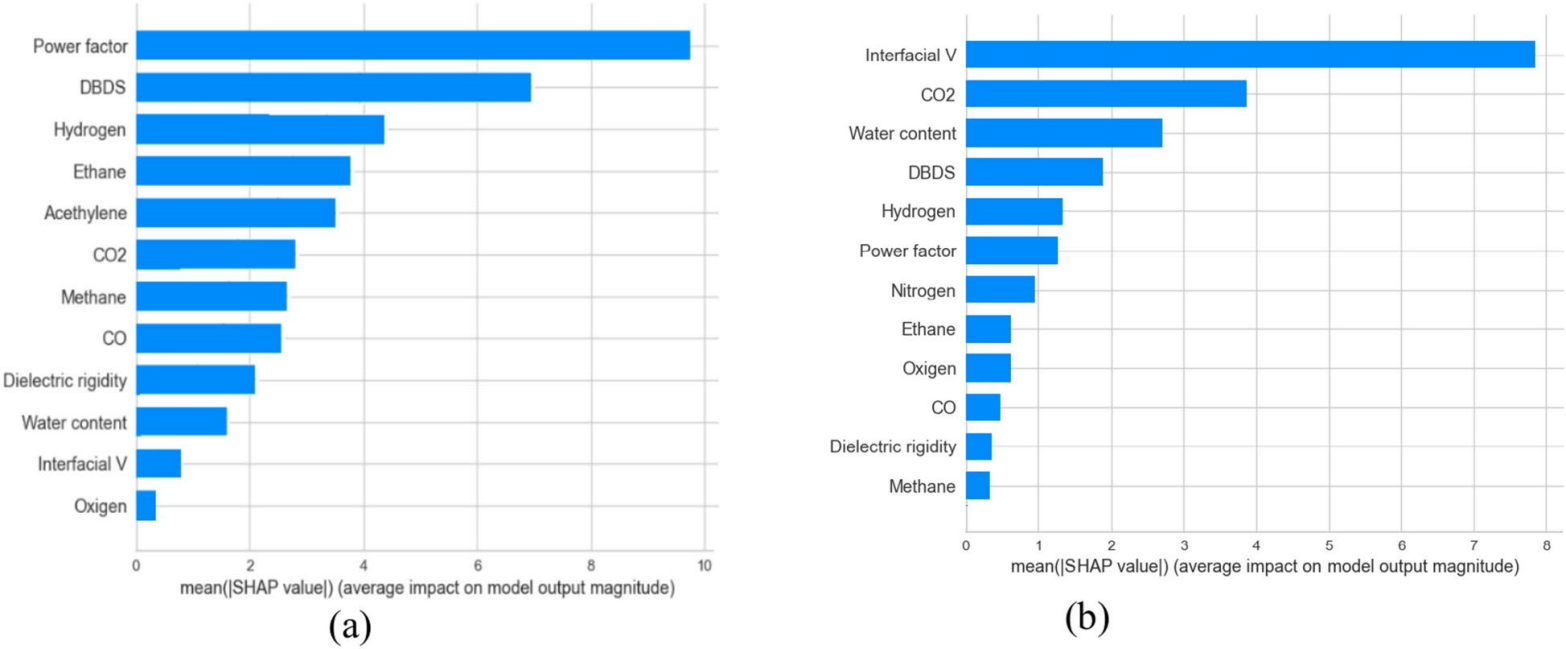
Feature importance according to the proposed model.

**Fig. 18:**
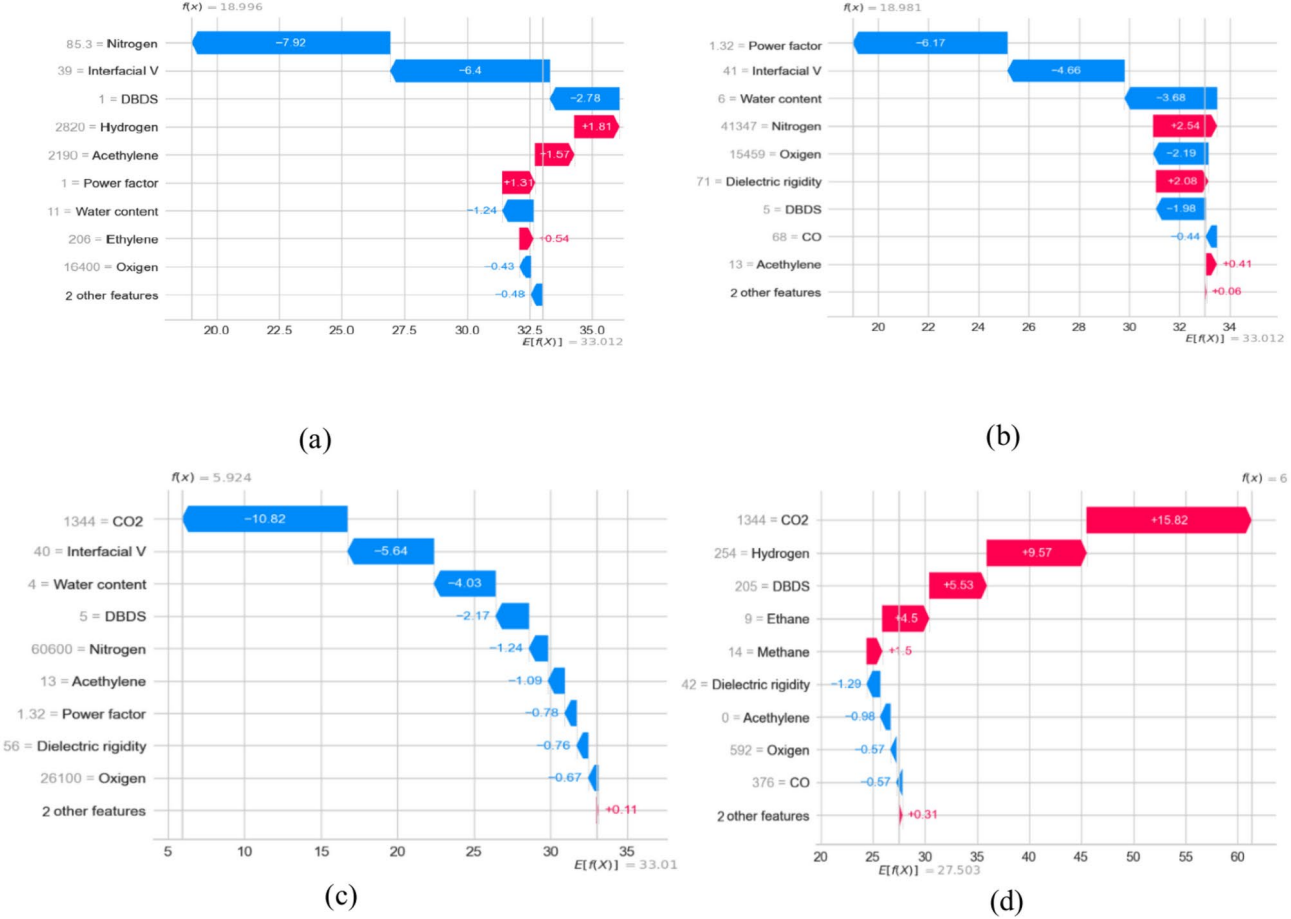
Waterfall plot according to the proposed model (**a**, **b**) according to THI (**c**, **d**) according to life expectation according to SHAP.

**Fig. 19:**
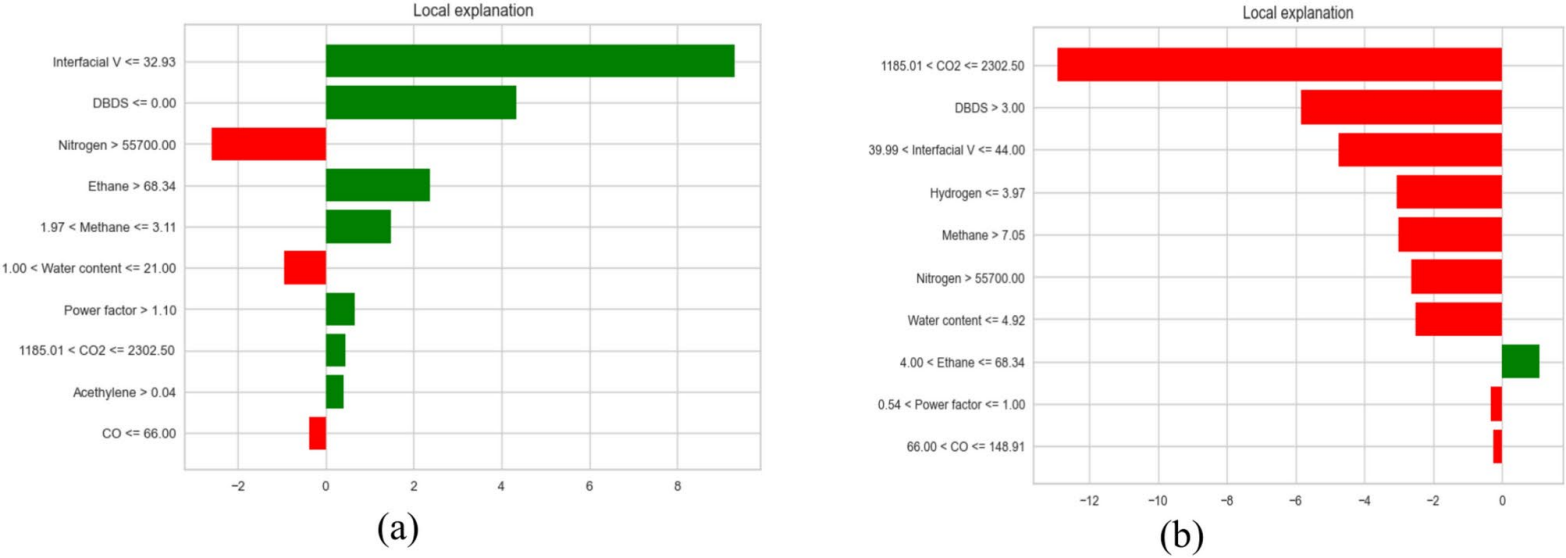
local explanation according to the proposed model (**a**) according to THI (**b**) according to life expectation according to LIME.

This section provides global and local explanations for the developed models. Knowing which features have a significant role in model prediction is crucial. Figure 17a, b shows the sorted list of features considered in the final THI and life expectation prediction, respectively. SHAP explainer utilized to calculate the feature importance in terms of dataset level. As shown in Fig. 17a. It is the most crucial feature in THI prediction. These features are essential from an engineering point of view. For example, in^32,33^, the authors concluded that CO_2_, hydrogen, Hydrogen, nitrogen are an essential indicator for the transformer health index. In addition Power factor, Methane, Co, Oxygen, and DielectricDBIS which has a less impact The same for life expectation in Fig. 17b, SHAP shows that CO_2_ and water content has the most impact on prediction, which is also confirmed in^34,35^, After analyzing the global explanation using SHAP, we need to ensure the importance of the suggested features at the instance level. We give two examples for each case (see Fig. 17a, b). It shows the waterfall plot. The values of the features were. These features contribute to getting the prediction of for health index as shown in Fig. 18a, b. The same is for Fig. 18c and d, which show the waterfall for life expectation prediction. From Fig. 14b, the engineer can visually measure the Value of each feature and its importance in the overall decision. As a result, the engineer can ensure the model makes the right decision. As shown in Fig. 18b, CO_2_ consider critical in specifying the importance of explainability in understanding the developed decisions, and they may not be enough for experts as they rely on a set of rules derived from their own experience and expertise when making decisions. To ensure the significance of the features, we utilized LIME to provide local explanation. Figure 19 shows local explanation according to the proposed model according to THI and life expectation. LIME give the same results as SHAP, which ensure the importance of the features in the overall decision.

**Fig. 20:**
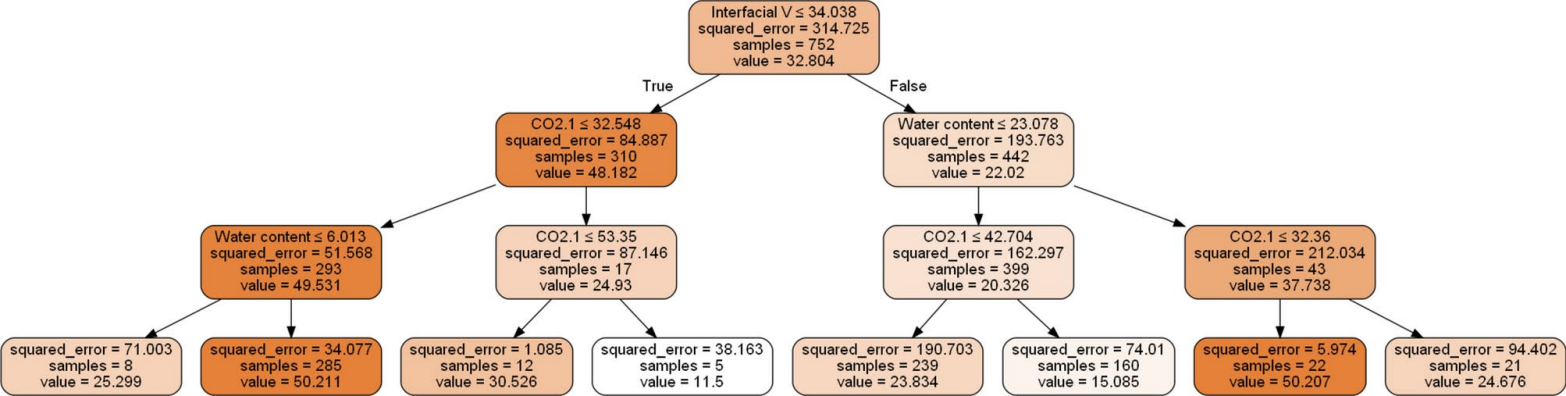
Decision tree for AXI (THI task).

**Fig. 21:**
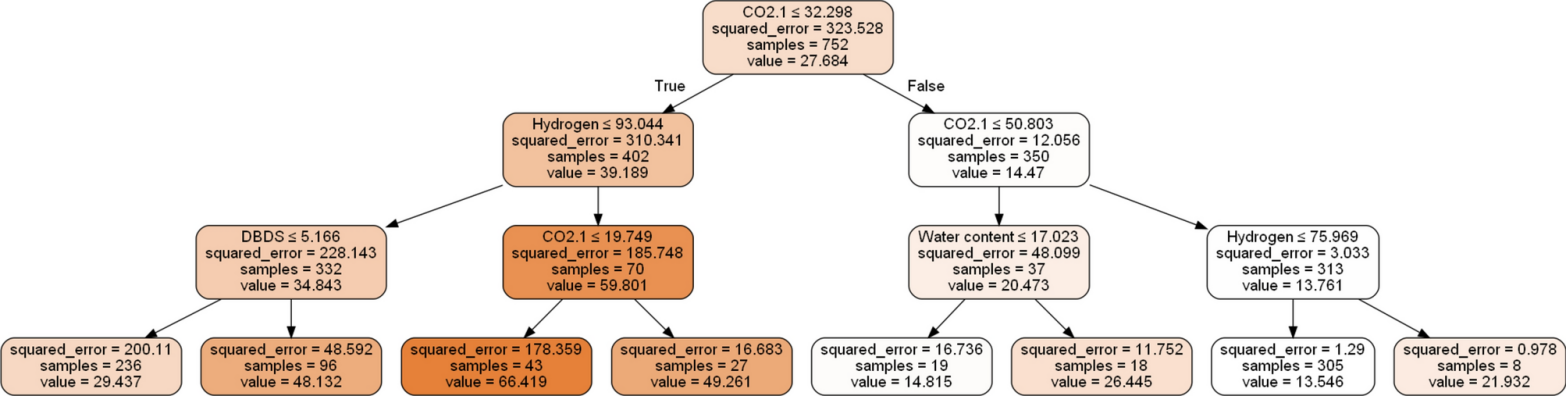
Decision tree for AXI (Timelife expectation task).

The LSTM_GRU model, which was the most accurate model, is considered the black box model and is challenging to extract rules from. Therefore, to provide the rules that the model depends on take that decision, we used a post-hoc explainable (Decision tree) model) to extract rules from the model. By following the path from the tree’s root to every leaf, it is straightforward to extract a set of rules from a decision tree. For example, Figs. 20 and 21 provide examples of decision tree regressors for the two tasks, respectively. Engineers can use these decision trees to collect the exact set of rules that the model uses, which can help them confirm their decisions and assist junior engineers in learning more about the decision-making process.

## Conclusion

Power transformers have a vital role in integrating renewable energy sources and improving the efficiency and reliability of smart grid systems. They facilitate the conversion, transmission, and distribution of power from diverse sources and help balance the load across the grid. To ensure their reliability and prevent unexpected outages, THI is a crucial indicator. This study was presented a new architecture called the Smart Electricity Monitoring System based on Fog Computing and Digital Twins (SEMS-FDT). This system monitors the health performance of transformers by measuring the THI rate in real-time.

The SEMS-FDT architecture was specifically designed for real-time observation of transformer health and performance. The study was investigated the use of ML models, including traditional and ensemble methods, to predict THI and LI. It explores the utilization of the entire set of features and optimized feature subsets for prediction. To enhance forecasting accuracy and achieve optimal performance, a novel multitasks LSTM_GRU model was proposed.

Experimental results demonstrated promising performance with MSE, MAE, MedAE, and R2 scores of 2.543, 0.13646, 0.0284, and 0.985, respectively. Additionally, the framework incorporated model explanations, including global explanations that provide insights based on the entire dataset and local explanations that offer instance-specific explanations. The integration of the proposed model and explainability features provides comprehensive outcomes for engineers regarding the model’s results.

## Data Availability

Data is available on request due to ethical restrictions. Contact with corresponding author to request the data.
